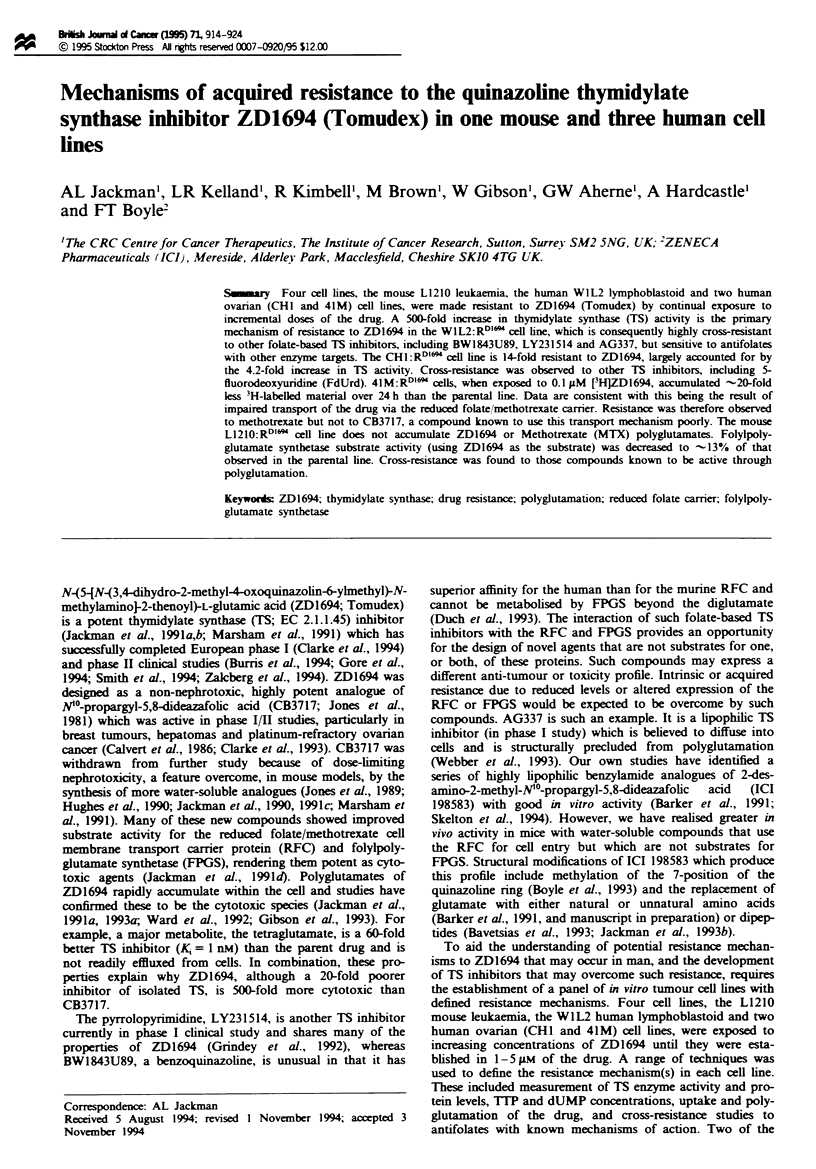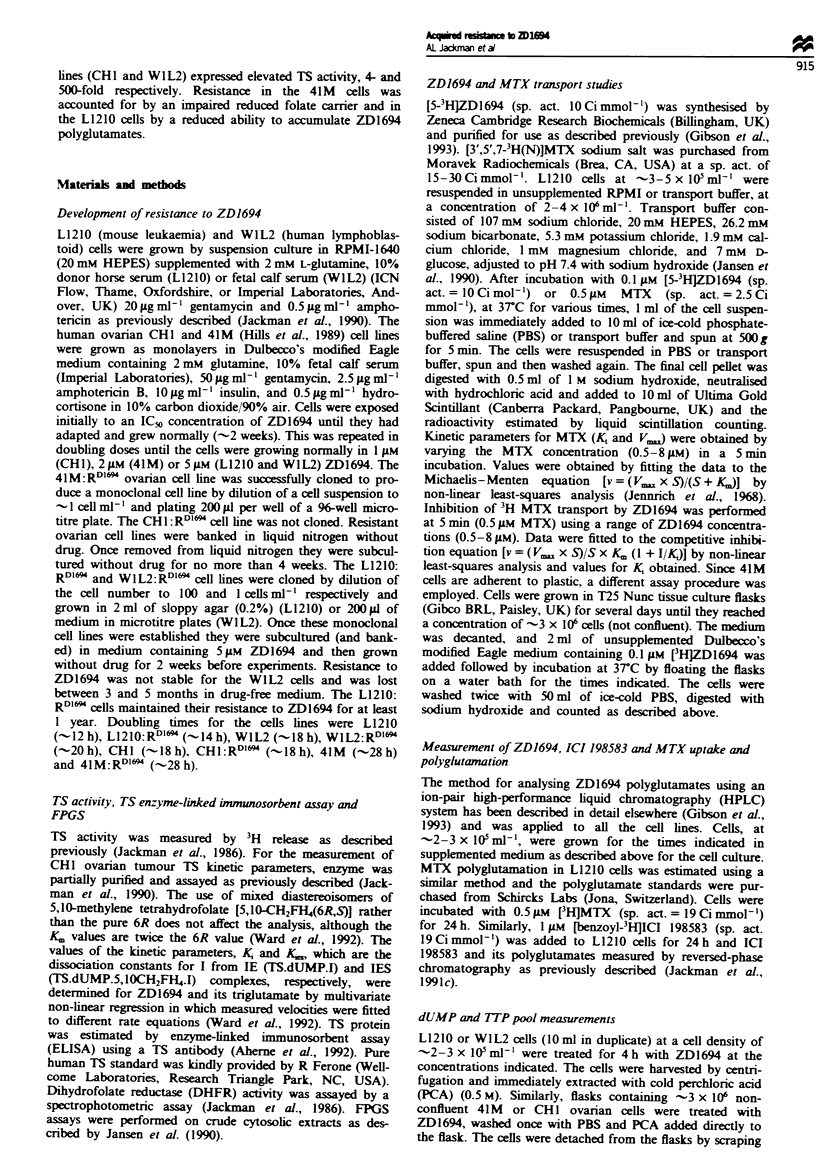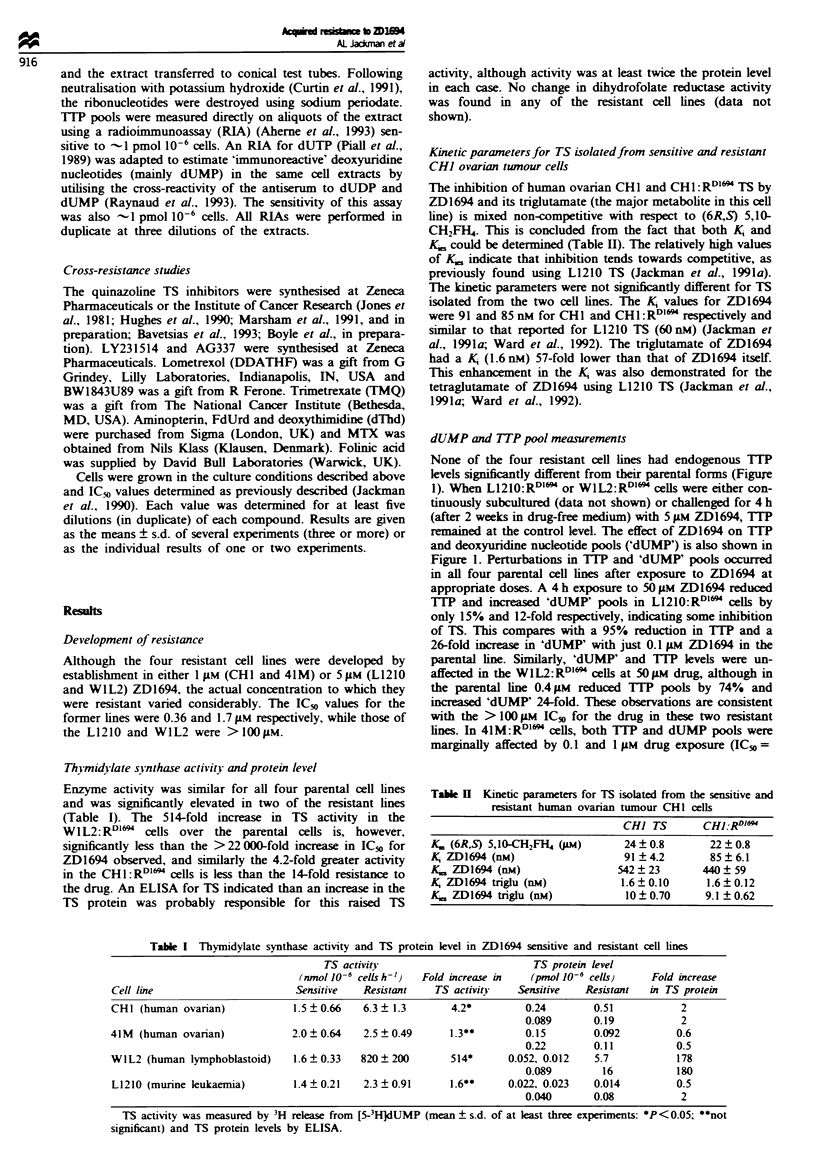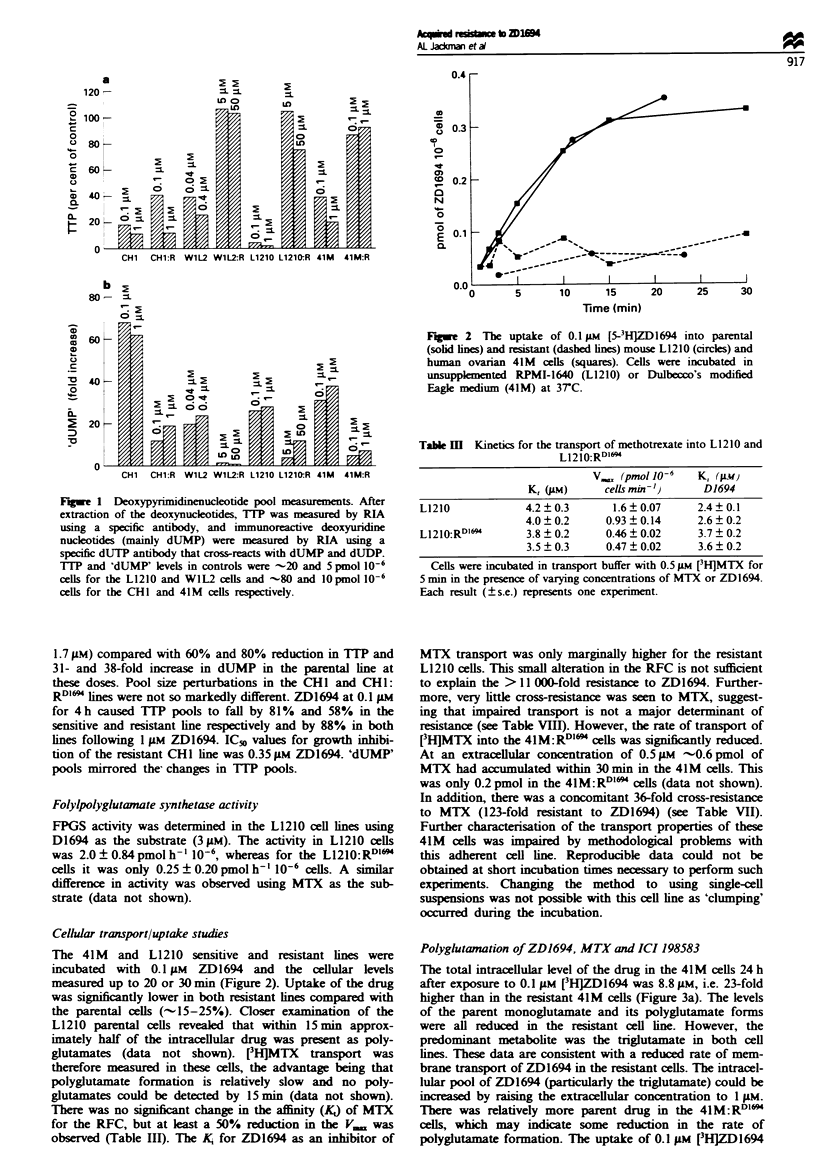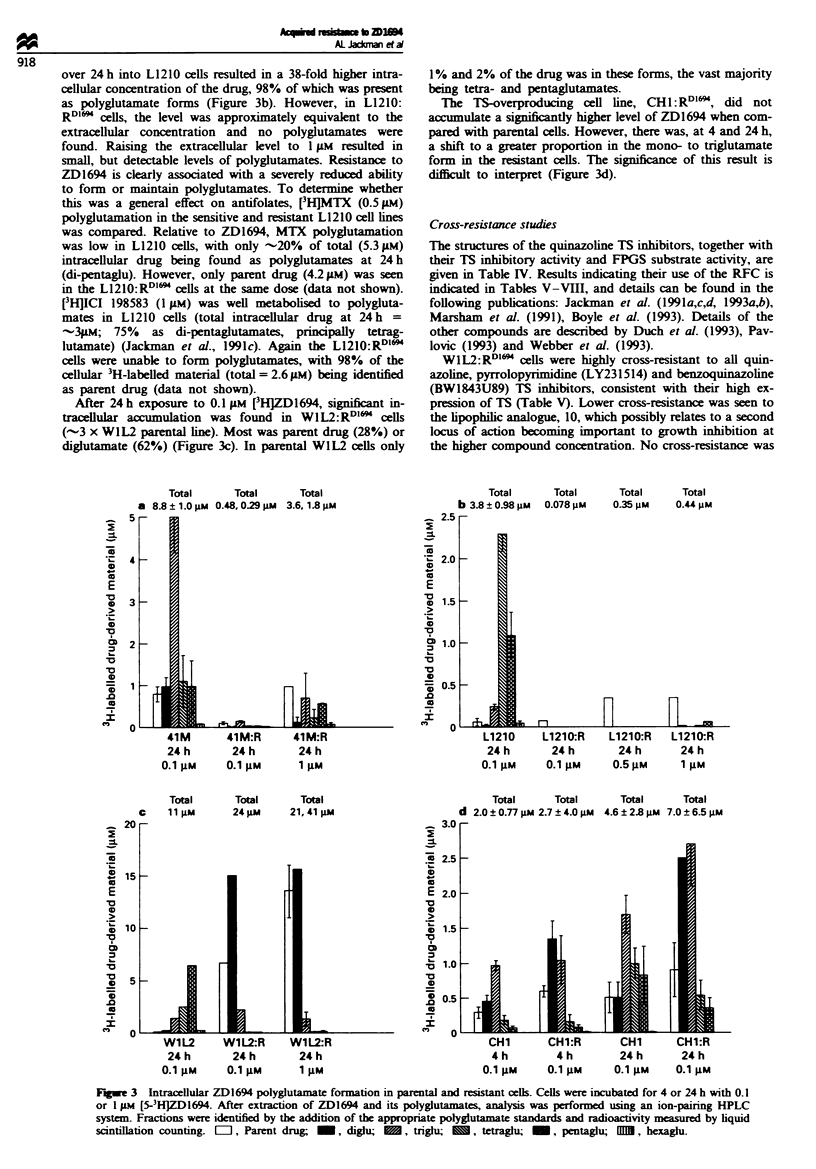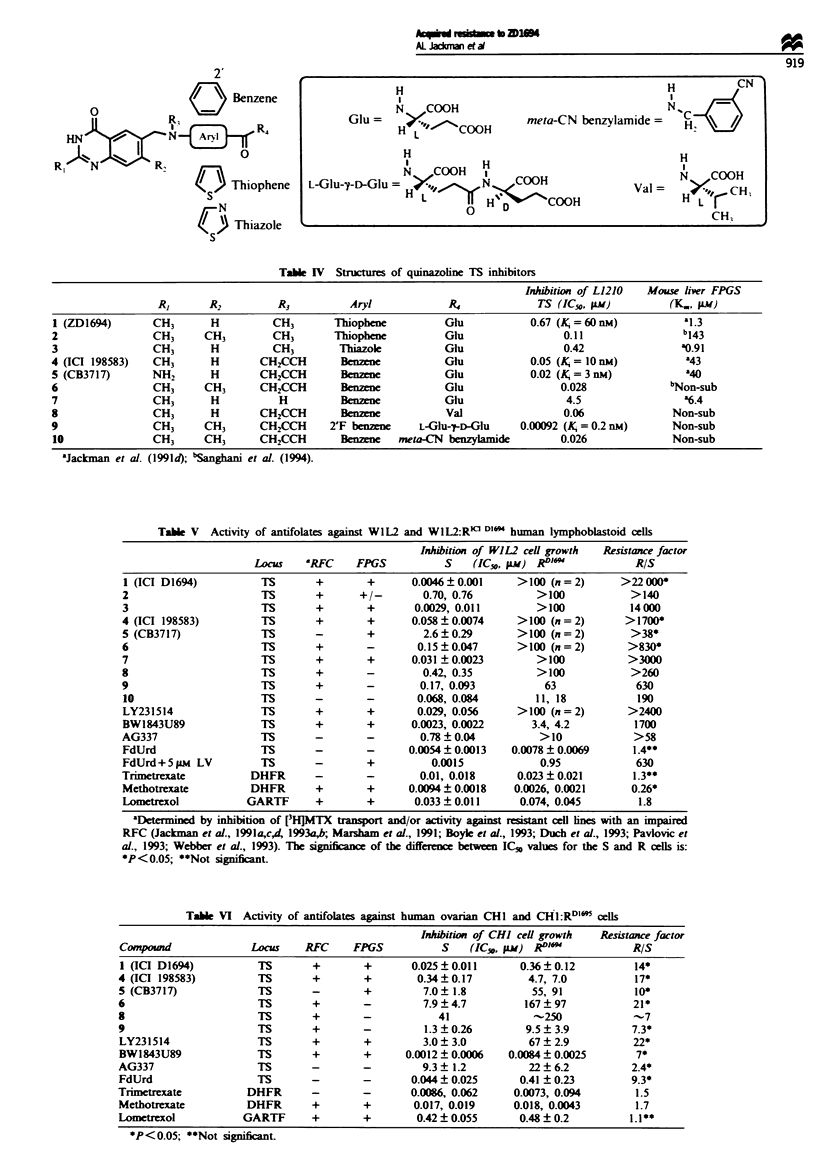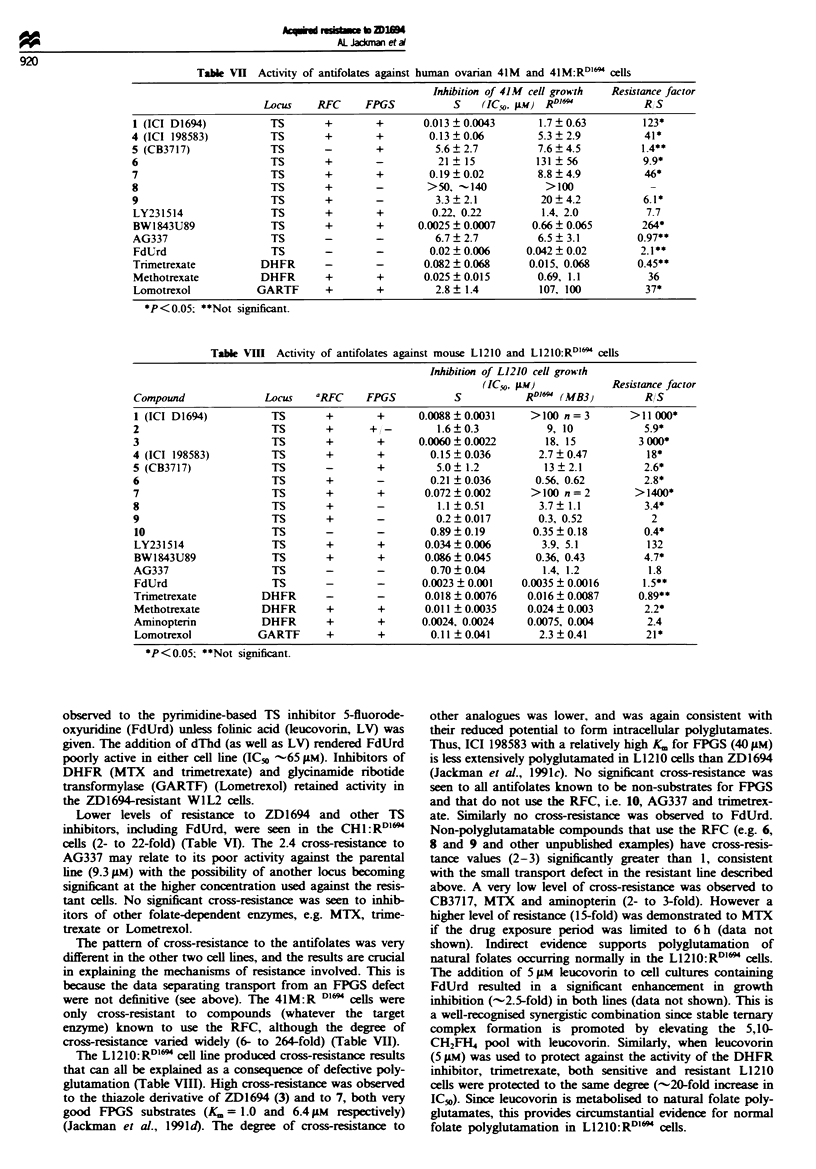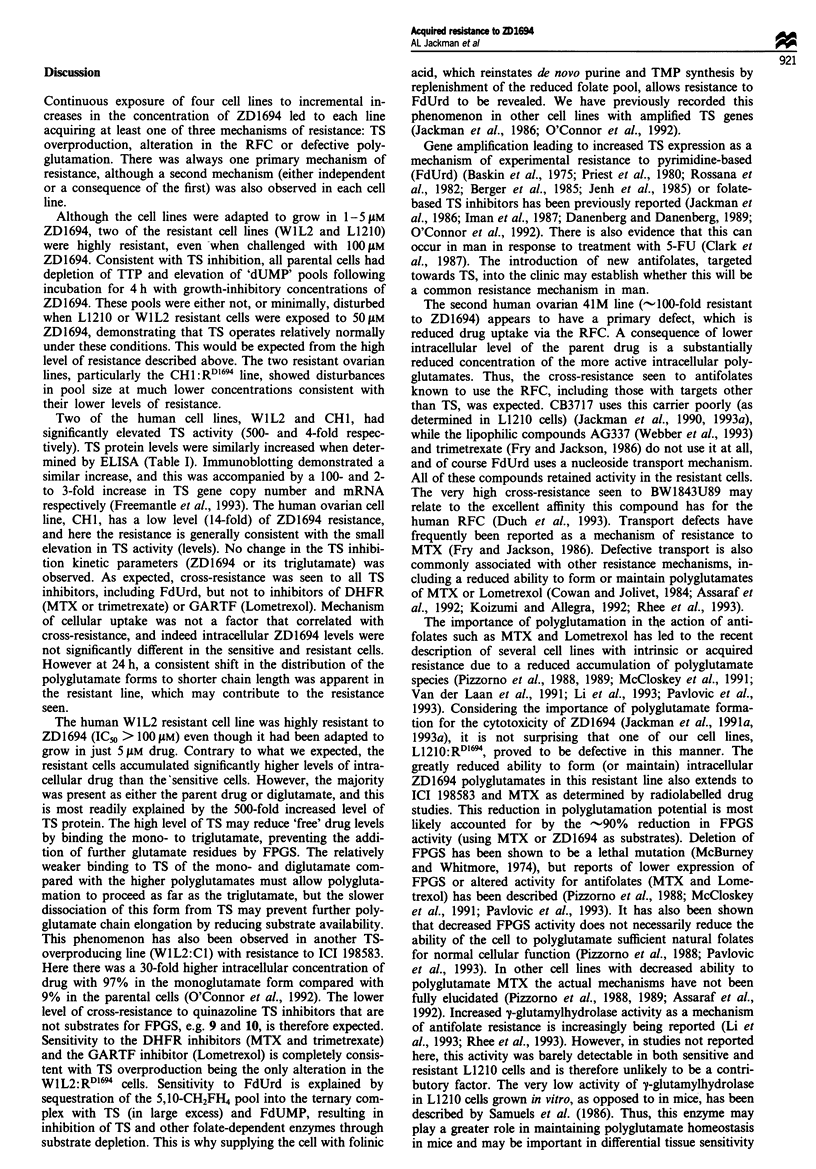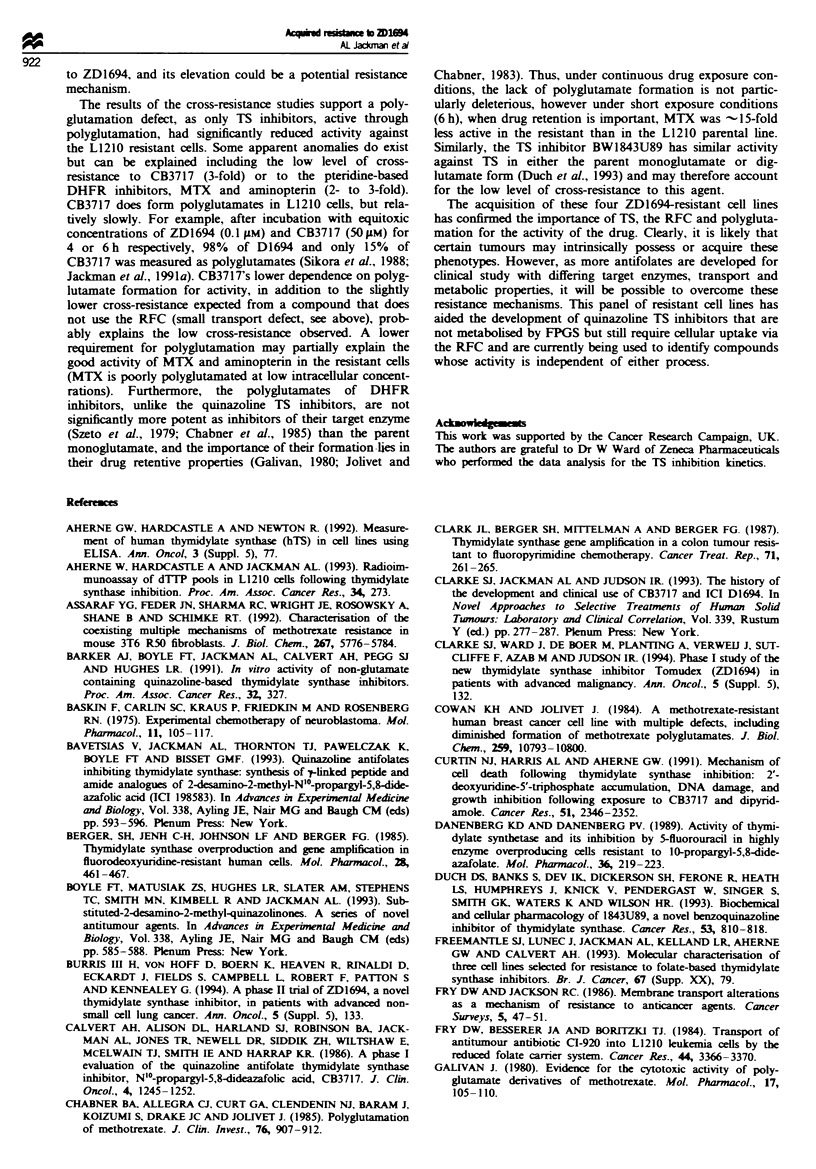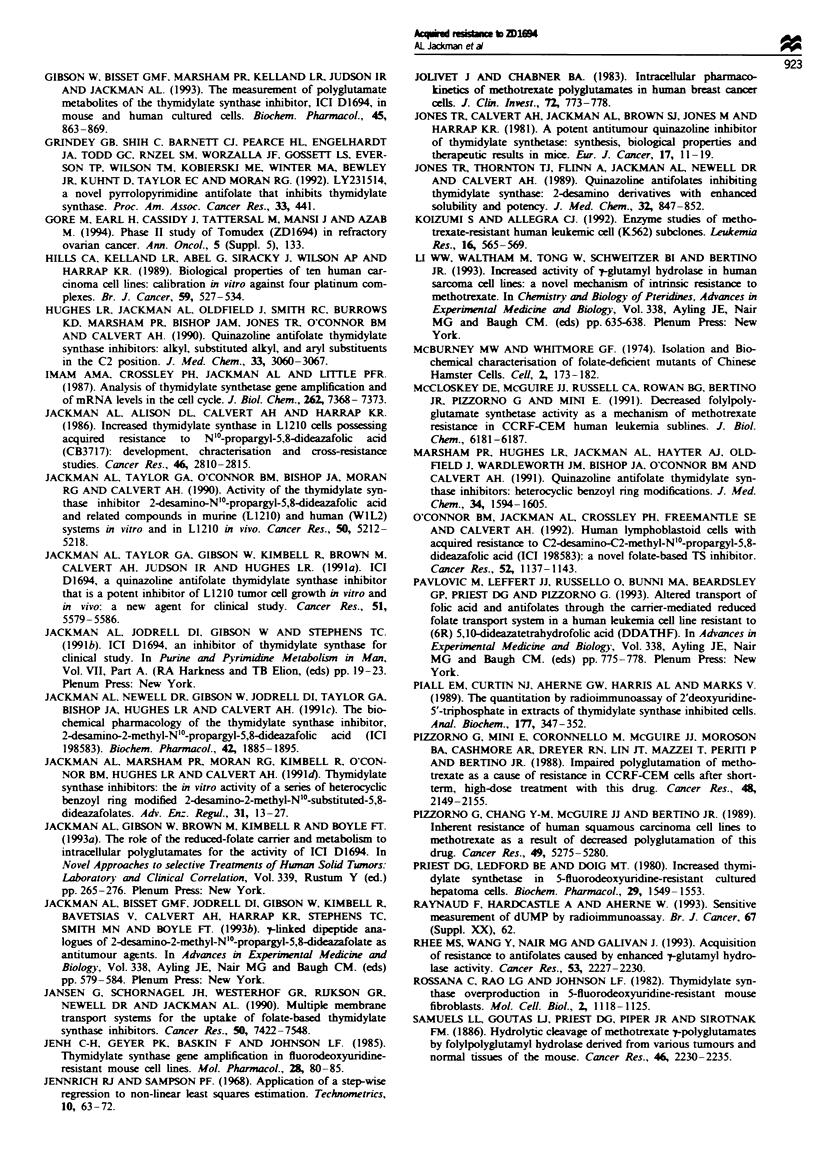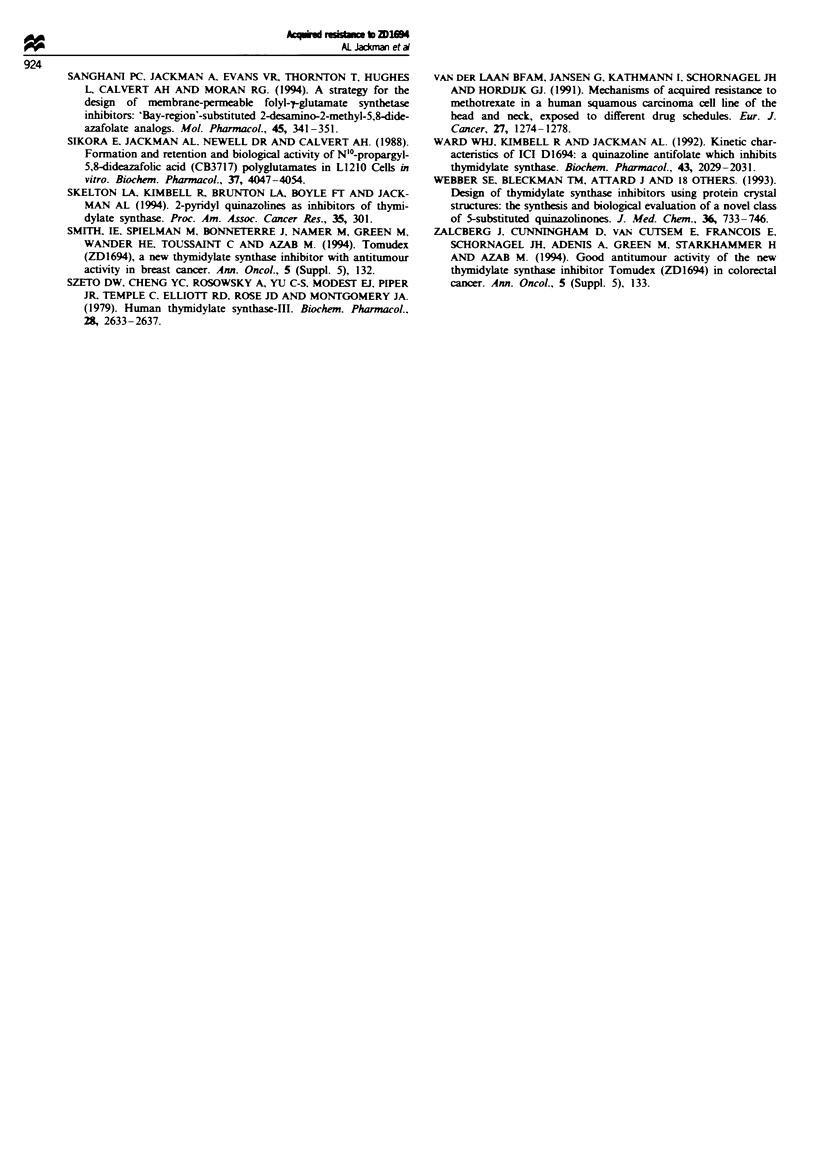# Mechanisms of acquired resistance to the quinazoline thymidylate synthase inhibitor ZD1694 (Tomudex) in one mouse and three human cell lines.

**DOI:** 10.1038/bjc.1995.178

**Published:** 1995-05

**Authors:** A. L. Jackman, L. R. Kelland, R. Kimbell, M. Brown, W. Gibson, G. W. Aherne, A. Hardcastle, F. T. Boyle

**Affiliations:** CRC Centre for Cancer Therapeutics, Institute of Cancer Research, Sutton, Surrey, UK.

## Abstract

Four cell lines, the mouse L1210 leukaemia, the human W1L2 lymphoblastoid and two human ovarian (CH1 and 41M) cell lines, were made resistant to ZD1694 (Tomudex) by continual exposure to incremental doses of the drug. A 500-fold increase in thymidylate synthase (TS) activity is the primary mechanism of resistance to ZD1694 in the W1L2:RD1694 cell line, which is consequently highly cross-resistant to other folate-based TS inhibitors, including BW1843U89, LY231514 and AG337, but sensitive to antifolates with other enzyme targets. The CH1:RD1694 cell line is 14-fold resistant to ZD1694, largely accounted for by the 4.2-fold increase in TS activity. Cross-resistance was observed to other TS inhibitors, including 5-fluorodeoxyuridine (FdUrd). 41M:RD1694 cells, when exposed to 0.1 microM [3H]ZD1694, accumulated approximately 20-fold less 3H-labelled material over 24 h than the parental line. Data are consistent with this being the result of impaired transport of the drug via the reduced folate/methotrexate carrier. Resistance was therefore observed to methotrexate but not to CB3717, a compound known to use this transport mechanism poorly. The mouse L1210:RD1694 cell line does not accumulate ZD1694 or Methotrexate (MTX) polyglutamates. Folylpolyglutamate synthetase substrate activity (using ZD1694 as the substrate) was decreased to approximately 13% of that observed in the parental line. Cross-resistance was found to those compounds known to be active through polyglutamation.


British Journal of Cancer (1995) 71, 914-924
© 1995 Stockton Press All rights reserved 0007-0920/95 $12.00

### Mechanisms of acquired resistance to the quinazoline thymidylate synthase inhibitor ZD1694 (Tomudex) in one mouse and three human cell lines

AL Jackman[1], LR Kelland[1], R Kimbell[1], M Brown[1], W Gibson[1], GW Aherne[1], A Hardcastle[1] and FT Boyle[2]

[1]*The CRC Centre for Cancer Therapeutics, The Institute of Cancer Research, Sutton, Surrey SM2 5NG, UK;* [2]*ZENECA Pharmaceuticals (ICI), Mereside, Alderley Park, Macclesfield, Cheshire SK10 4TG UK.*

**Summary** Four cell lines, the mouse L1210 leukaemia, the human W1L2 lymphoblastoid and two human ovarian (CH1 and 41M) cell lines, were made resistant to ZD1694 (Tomudex) by continual exposure to incremental doses of the drug. A 500-fold increase in thymidylate synthase (TS) activity is the primary mechanism of resistance to ZD1694 in the W1L2:$R^{D1694}$ cell line, which is consequently highly cross-resistant to other folate-based TS inhibitors, including BW1843U89, LY231514 and AG337, but sensitive to antifolates with other enzyme targets. The CH1:$R^{D1694}$ cell line is 14-fold resistant to ZD1694, largely accounted for by the 4.2-fold increase in TS activity. Cross-resistance was observed to other TS inhibitors, including 5-fluorodeoxyuridine (FdUrd). 41M:$R^{D1694}$ cells, when exposed to 0.1 μM [$^3$H]ZD1694, accumulated ~20-fold less $^3$H-labelled material over 24 h than the parental line. Data are consistent with this being the result of impaired transport of the drug via the reduced folate/methotrexate carrier. Resistance was therefore observed to methotrexate but not to CB3717, a compound known to use this transport mechanism poorly. The mouse L1210:$R^{D1694}$ cell line does not accumulate ZD1694 or Methotrexate (MTX) polyglutamates. Folylpolyglutamate synthetase substrate activity (using ZD1694 as the substrate) was decreased to ~13% of that observed in the parental line. Cross-resistance was found to those compounds known to be active through polyglutamation.

**Keywords:** ZD1694; thymidylate synthase; drug resistance; polyglutamation; reduced folate carrier; folylpolyglutamate synthetase

*N*-(5-[*N*-(3,4-dihydro-2-methyl-4-oxoquinazolin-6-ylmethyl)-*N*-methylamino]-2-thenoyl)-L-glutamic acid (ZD1694; Tomudex) is a potent thymidylate synthase (TS; EC 2.1.1.45) inhibitor (Jackman *et al.*, 1991*a,b*; Marsham *et al.*, 1991) which has successfully completed European phase I (Clarke *et al.*, 1994) and phase II clinical studies (Burris *et al.*, 1994; Gore *et al.*, 1994; Smith *et al.*, 1994; Zalcberg *et al.*, 1994). ZD1694 was designed as a non-nephrotoxic, highly potent analogue of $N^{10}$-propargyl-5,8-dideazafolic acid (CB3717; Jones *et al.*, 1981) which was active in phase I/II studies, particularly in breast tumours, hepatomas and platinum-refractory ovarian cancer (Calvert *et al.*, 1986; Clarke *et al.*, 1993). CB3717 was withdrawn from further study because of dose-limiting nephrotoxicity, a feature overcome, in mouse models, by the synthesis of more water-soluble analogues (Jones *et al.*, 1989; Hughes *et al.*, 1990; Jackman *et al.*, 1990, 1991*c*; Marsham *et al.*, 1991). Many of these new compounds showed improved substrate activity for the reduced folate/methotrexate cell membrane transport carrier protein (RFC) and folylpolyglutamate synthetase (FPGS), rendering them potent as cytotoxic agents (Jackman *et al.*, 1991*d*). Polyglutamates of ZD1694 rapidly accumulate within the cell and studies have confirmed these to be the cytotoxic species (Jackman *et al.*, 1991*a*, 1993*a*; Ward *et al.*, 1992; Gibson *et al.*, 1993). For example, a major metabolite, the tetraglutamate, is a 60-fold better TS inhibitor ($K_i$ = 1 nM) than the parent drug and is not readily effluxed from cells. In combination, these properties explain why ZD1694, although a 20-fold poorer inhibitor of isolated TS, is 500-fold more cytotoxic than CB3717.

The pyrrolopyrimidine, LY231514, is another TS inhibitor currently in phase I clinical study and shares many of the properties of ZD1694 (Grindey *et al.*, 1992), whereas BW1843U89, a benzoquinazoline, is unusual in that it has superior affinity for the human than for the murine RFC and cannot be metabolised by FPGS beyond the diglutamate (Duch *et al.*, 1993). The interaction of such folate-based TS inhibitors with the RFC and FPGS provides an opportunity for the design of novel agents that are not substrates for one, or both, of these proteins. Such compounds may express a different anti-tumour or toxicity profile. Intrinsic or acquired resistance due to reduced levels or altered expression of the RFC or FPGS would be expected to be overcome by such compounds. AG337 is such an example. It is a lipophilic TS inhibitor (in phase I study) which is believed to diffuse into cells and is structurally precluded from polyglutamation (Webber *et al.*, 1993). Our own studies have identified a series of highly lipophilic benzylamide analogues of 2-desamino-2-methyl-$N^{10}$-propargyl-5,8-dideazafolic acid (ICI 198583) with good *in vitro* activity (Barker *et al.*, 1991; Skelton *et al.*, 1994). However, we have realised greater *in vivo* activity in mice with water-soluble compounds that use the RFC for cell entry but which are not substrates for FPGS. Structural modifications of ICI 198583 which produce this profile include methylation of the 7-position of the quinazoline ring (Boyle *et al.*, 1993) and the replacement of glutamate with either natural or unnatural amino acids (Barker *et al.*, 1991, and manuscript in preparation) or dipeptides (Bavetsias *et al.*, 1993; Jackman *et al.*, 1993*b*).

To aid the understanding of potential resistance mechanisms to ZD1694 that may occur in man, and the development of TS inhibitors that may overcome such resistance, requires the establishment of a panel of *in vitro* tumour cell lines with defined resistance mechanisms. Four cell lines, the L1210 mouse leukaemia, the W1L2 human lymphoblastoid and two human ovarian (CH1 and 41M) cell lines, were exposed to increasing concentrations of ZD1694 until they were established in 1-5 μM of the drug. A range of techniques was used to define the resistance mechanism(s) in each cell line. These included measurement of TS enzyme activity and protein levels, TTP and dUMP concentrations, uptake and polyglutamation of the drug, and cross-resistance studies to antifolates with known mechanisms of action. Two of the

Correspondence: AL Jackman
Received 5 August 1994; revised 1 November 1994; accepted 3 November 1994

lines (CH1 and W1L2) expressed elevated TS activity, 4- and 500-fold respectively. Resistance in the 41M cells was accounted for by an impaired reduced folate carrier and in the L1210 cells by a reduced ability to accumulate ZD1694 polyglutamates.

### Materials and methods

*Development of resistance to ZD1694*

L1210 (mouse leukaemia) and W1L2 (human lymphoblastoid) cells were grown by suspension culture in RPMI-1640 (20 mM HEPES) supplemented with 2 mM L-glutamine, 10% donor horse serum (L1210) or fetal calf serum (W1L2) (ICN Flow, Thame, Oxfordshire, or Imperial Laboratories, Andover, UK) 20 $\mu g\ ml^{-1}$ gentamycin and 0.5 $\mu g\ ml^{-1}$ amphotericin as previously described (Jackman *et al.*, 1990). The human ovarian CH1 and 41M (Hills *et al.*, 1989) cell lines were grown as monolayers in Dulbecco's modified Eagle medium containing 2 mM glutamine, 10% fetal calf serum (Imperial Laboratories), 50 $\mu g\ ml^{-1}$ gentamycin, 2.5 $\mu g\ ml^{-1}$ amphotericin B, 10 $\mu g\ ml^{-1}$ insulin, and 0.5 $\mu g\ ml^{-1}$ hydrocortisone in 10% carbon dioxide/90% air. Cells were exposed initially to an $IC_{50}$ concentration of ZD1694 until they had adapted and grew normally (~2 weeks). This was repeated in doubling doses until the cells were growing normally in 1 $\mu$M (CH1), 2 $\mu$M (41M) or 5 $\mu$M (L1210 and W1L2) ZD1694. The 41M:$R^{D1694}$ ovarian cell line was successfully cloned to produce a monoclonal cell line by dilution of a cell suspension to ~1 cell $ml^{-1}$ and plating 200 $\mu$l per well of a 96-well microtitre plate. The CH1:$R^{D1694}$ cell line was not cloned. Resistant ovarian cell lines were banked in liquid nitrogen without drug. Once removed from liquid nitrogen they were subcultured without drug for no more than 4 weeks. The L1210:$R^{D1694}$ and W1L2:$R^{D1694}$ cell lines were cloned by dilution of the cell number to 100 and 1 cells $ml^{-1}$ respectively and grown in 2 ml of sloppy agar (0.2%) (L1210) or 200 $\mu$l of medium in microtitre plates (W1L2). Once these monoclonal cell lines were established they were subcultured (and banked) in medium containing 5 $\mu$M ZD1694 and then grown without drug for 2 weeks before experiments. Resistance to ZD1694 was not stable for the W1L2 cells and was lost between 3 and 5 months in drug-free medium. The L1210:$R^{D1694}$ cells maintained their resistance to ZD1694 for at least 1 year. Doubling times for the cells lines were L1210 (~12 h), L1210:$R^{D1694}$ (~14 h), W1L2 (~18 h), W1L2:$R^{D1694}$ (~20 h), CH1 (~18 h), CH1:$R^{D1694}$ (~18 h), 41M (~28 h) and 41M:$R^{D1694}$ (~28 h).

*TS activity, TS enzyme-linked immunosorbent assay and FPGS*

TS activity was measured by $^3H$ release as described previously (Jackman *et al.*, 1986). For the measurement of CH1 ovarian tumour TS kinetic parameters, enzyme was partially purified and assayed as previously described (Jackman *et al.*, 1990). The use of mixed diastereoisomers of 5,10-methylene tetrahydrofolate [5,10-$CH_2FH_4$(6*R,S*)] rather than the pure 6*R* does not affect the analysis, although the $K_m$ values are twice the 6*R* value (Ward *et al.*, 1992). The values of the kinetic parameters, $K_i$ and $K_{ies}$, which are the dissociation constants for I from IE (TS.dUMP.I) and IES (TS.dUMP.5,10$CH_2FH_4$.I) complexes, respectively, were determined for ZD1694 and its triglutamate by multivariate non-linear regression in which measured velocities were fitted to different rate equations (Ward *et al.*, 1992). TS protein was estimated by enzyme-linked immunosorbent assay (ELISA) using a TS antibody (Aherne *et al.*, 1992). Pure human TS standard was kindly provided by R Ferone (Wellcome Laboratories, Research Triangle Park, NC, USA). Dihydrofolate reductase (DHFR) activity was assayed by a spectrophotometric assay (Jackman *et al.*, 1986). FPGS assays were performed on crude cytosolic extracts as described by Jansen *et al.* (1990).

*ZD1694 and MTX transport studies*

[5-$^3$H]ZD1694 (sp. act. 10 Ci $mmol^{-1}$) was synthesised by Zeneca Cambridge Research Biochemicals (Billingham, UK) and purified for use as described previously (Gibson *et al.*, 1993). [3′,5′,7-$^3$H(N)]MTX sodium salt was purchased from Moravek Radiochemicals (Brea, CA, USA) at a sp. act. of 15–30 Ci $mmol^{-1}$. L1210 cells at ~$3-5\times10^5$ $ml^{-1}$ were resuspended in unsupplemented RPMI or transport buffer, at a concentration of $2-4\times10^6$ $ml^{-1}$. Transport buffer consisted of 107 mM sodium chloride, 20 mM HEPES, 26.2 mM sodium bicarbonate, 5.3 mM potassium chloride, 1.9 mM calcium chloride, 1 mM magnesium chloride, and 7 mM D-glucose, adjusted to pH 7.4 with sodium hydroxide (Jansen *et al.*, 1990). After incubation with 0.1 $\mu$M [5-$^3$H]ZD1694 (sp. act. = 10 Ci $mol^{-1}$) or 0.5 $\mu$M MTX (sp. act. = 2.5 Ci $mmol^{-1}$), at 37°C for various times, 1 ml of the cell suspension was immediately added to 10 ml of ice-cold phosphate-buffered saline (PBS) or transport buffer and spun at 500 *g* for 5 min. The cells were resuspended in PBS or transport buffer, spun and then washed again. The final cell pellet was digested with 0.5 ml of 1 M sodium hydroxide, neutralised with hydrochloric acid and added to 10 ml of Ultima Gold Scintillant (Canberra Packard, Pangbourne, UK) and the radioactivity estimated by liquid scintillation counting. Kinetic parameters for MTX ($K_t$ and $V_{max}$) were obtained by varying the MTX concentration (0.5–8 $\mu$M) in a 5 min incubation. Values were obtained by fitting the data to the Michaelis–Menten equation [$v = (V_{max} \times S)/(S + K_m)$] by non-linear least-squares analysis (Jennrich *et al.*, 1968). Inhibition of $^3$H MTX transport by ZD1694 was performed at 5 min (0.5 $\mu$M MTX) using a range of ZD1694 concentrations (0.5–8 $\mu$M). Data were fitted to the competitive inhibition equation [$v = (V_{max} \times S)/S \times K_m\ (1 + I/K_i)$] by non-linear least-squares analysis and values for $K_i$ obtained. Since 41M cells are adherent to plastic, a different assay procedure was employed. Cells were grown in T25 Nunc tissue culture flasks (Gibco BRL, Paisley, UK) for several days until they reached a concentration of ~$3\times10^6$ cells (not confluent). The medium was decanted, and 2 ml of unsupplemented Dulbecco's modified Eagle medium containing 0.1 $\mu$M [$^3$H]ZD1694 was added followed by incubation at 37°C by floating the flasks on a water bath for the times indicated. The cells were washed twice with 50 ml of ice-cold PBS, digested with sodium hydroxide and counted as described above.

*Measurement of ZD1694, ICI 198583 and MTX uptake and polyglutamation*

The method for analysing ZD1694 polyglutamates using an ion-pair high-performance liquid chromatography (HPLC) system has been described in detail elsewhere (Gibson *et al.*, 1993) and was applied to all the cell lines. Cells, at ~$2-3\times10^5$ $ml^{-1}$, were grown for the times indicated in supplemented medium as described above for the cell culture. MTX polyglutamation in L1210 cells was estimated using a similar method and the polyglutamate standards were purchased from Schircks Labs (Jona, Switzerland). Cells were incubated with 0.5 $\mu$M [$^3$H]MTX (sp. act. = 19 Ci $mmol^{-1}$) for 24 h. Similarly, 1 $\mu$M [benzoyl-$^3$H]ICI 198583 (sp. act. 19 Ci $mmol^{-1}$) was added to L1210 cells for 24 h and ICI 198583 and its polyglutamates measured by reversed-phase chromatography as previously described (Jackman *et al.*, 1991*c*).

*dUMP and TTP pool measurements*

L1210 or W1L2 cells (10 ml in duplicate) at a cell density of ~$2-3\times10^5$ $ml^{-1}$ were treated for 4 h with ZD1694 at the concentrations indicated. The cells were harvested by centrifugation and immediately extracted with cold perchloric acid (PCA) (0.5 M). Similarly, flasks containing ~$3\times10^6$ non-confluent 41M or CH1 ovarian cells were treated with ZD1694, washed once with PBS and PCA added directly to the flask. The cells were detached from the flasks by scraping and the extract transferred to conical test tubes. Following neutralisation with potassium hydroxide (Curtin *et al.*, 1991), the ribonucleotides were destroyed using sodium periodate. TTP pools were measured directly on aliquots of the extract using a radioimmunoassay (RIA) (Aherne *et al.*, 1993) sensitive to ~1 pmol $10^{-6}$ cells. An RIA for dUTP (Piall *et al.*, 1989) was adapted to estimate 'immunoreactive' deoxyuridine nucleotides (mainly dUMP) in the same cell extracts by utilising the cross-reactivity of the antiserum to dUDP and dUMP (Raynaud *et al.*, 1993). The sensitivity of this assay was also ~1 pmol $10^{-6}$ cells. All RIAs were performed in duplicate at three dilutions of the extracts.

*Cross-resistance studies*

The quinazoline TS inhibitors were synthesised at Zeneca Pharmaceuticals or the Institute of Cancer Research (Jones *et al.*, 1981; Hughes *et al.*, 1990; Marsham *et al.*, 1991, and in preparation; Bavetsias *et al.*, 1993; Boyle *et al.*, in preparation). LY231514 and AG337 were synthesised at Zeneca Pharmaceuticals. Lometrexol (DDATHF) was a gift from G Grindey, Lilly Laboratories, Indianapolis, IN, USA and BW1843U89 was a gift from R Ferone. Trimetrexate (TMQ) was a gift from The National Cancer Institute (Bethesda, MD, USA). Aminopterin, FdUrd and deoxythimidine (dThd) were purchased from Sigma (London, UK) and MTX was obtained from Nils Klass (Klausen, Denmark). Folinic acid was supplied by David Bull Laboratories (Warwick, UK).

Cells were grown in the culture conditions described above and $IC_{50}$ values determined as previously described (Jackman *et al.*, 1990). Each value was determined for at least five dilutions (in duplicate) of each compound. Results are given as the means ± s.d. of several experiments (three or more) or as the individual results of one or two experiments.

### Results

*Development of resistance*

Although the four resistant cell lines were developed by establishment in either 1 μM (CH1 and 41M) or 5 μM (L1210 and W1L2) ZD1694, the actual concentration to which they were resistant varied considerably. The $IC_{50}$ values for the former lines were 0.36 and 1.7 μM respectively, while those of the L1210 and W1L2 were >100 μM.

*Thymidylate synthase activity and protein level*

Enzyme activity was similar for all four parental cell lines and was significantly elevated in two of the resistant lines (Table I). The 514-fold increase in TS activity in the W1L2:$R^{D1694}$ cells over the parental cells is, however, significantly less than the >22 000-fold increase in $IC_{50}$ for ZD1694 observed, and similarly the 4.2-fold greater activity in the CH1:$R^{D1694}$ cells is less than the 14-fold resistance to the drug. An ELISA for TS indicated than an increase in the TS protein was probably responsible for this raised TS activity, although activity was at least twice the protein level in each case. No change in dihydrofolate reductase activity was found in any of the resistant cell lines (data not shown).

*Kinetic parameters for TS isolated from sensitive and resistant CH1 ovarian tumour cells*

The inhibition of human ovarian CH1 and CH1:$R^{D1694}$ TS by ZD1694 and its triglutamate (the major metabolite in this cell line) is mixed non-competitive with respect to (6*R*,*S*) 5,10-$CH_2FH_4$. This is concluded from the fact that both $K_i$ and $K_{ies}$ could be determined (Table II). The relatively high values of $K_{ies}$ indicate that inhibition tends towards competitive, as previously found using L1210 TS (Jackman *et al.*, 1991*a*). The kinetic parameters were not significantly different for TS isolated from the two cell lines. The $K_i$ values for ZD1694 were 91 and 85 nM for CH1 and CH1:$R^{D1694}$ respectively and similar to that reported for L1210 TS (60 nM) (Jackman *et al.*, 1991*a*; Ward *et al.*, 1992). The triglutamate of ZD1694 had a $K_i$ (1.6 nM) 57-fold lower than that of ZD1694 itself. This enhancement in the $K_i$ was also demonstrated for the tetraglutamate of ZD1694 using L1210 TS (Jackman *et al.*, 1991*a*; Ward *et al.*, 1992).

*dUMP and TTP pool measurements*

None of the four resistant cell lines had endogenous TTP levels significantly different from their parental forms (Figure 1). When L1210:$R^{D1694}$ or W1L2:$R^{D1694}$ cells were either continuously subcultured (data not shown) or challenged for 4 h (after 2 weeks in drug-free medium) with 5 μM ZD1694, TTP remained at the control level. The effect of ZD1694 on TTP and deoxyuridine nucleotide pools ('dUMP') is also shown in Figure 1. Perturbations in TTP and 'dUMP' pools occurred in all four parental cell lines after exposure to ZD1694 at appropriate doses. A 4 h exposure to 50 μM ZD1694 reduced TTP and increased 'dUMP' pools in L1210:$R^{D1694}$ cells by only 15% and 12-fold respectively, indicating some inhibition of TS. This compares with a 95% reduction in TTP and a 26-fold increase in 'dUMP' with just 0.1 μM ZD1694 in the parental line. Similarly, 'dUMP' and TTP levels were unaffected in the W1L2:$R^{D1694}$ cells at 50 μM drug, although in the parental line 0.4 μM reduced TTP pools by 74% and increased 'dUMP' 24-fold. These observations are consistent with the >100 μM $IC_{50}$ for the drug in these two resistant lines. In 41M:$R^{D1694}$ cells, both TTP and dUMP pools were marginally affected by 0.1 and 1 μM drug exposure ($IC_{50}$ =

**Table II** Kinetic parameters for TS isolated from the sensitive and resistant human ovarian tumour CH1 cells

| | *CH1 TS* | *CH1:$R^{D1694}$* |
|---|---|---|
| $K_m$ (6*R*,*S*) 5,10-$CH_2FH_4$ (μM) | 24 ± 0.8 | 22 ± 0.8 |
| $K_i$ ZD1694 (nM) | 91 ± 4.2 | 85 ± 6.1 |
| $K_{ies}$ ZD1694 (nM) | 542 ± 23 | 440 ± 59 |
| $K_i$ ZD1694 triglu (nM) | 1.6 ± 0.10 | 1.6 ± 0.12 |
| $K_{ies}$ ZD1694 triglu (nM) | 10 ± 0.70 | 9.1 ± 0.62 |

**Table I** Thymidylate synthase activity and TS protein level in ZD1694 sensitive and resistant cell lines

| *Cell line* | *TS activity (nmol $10^{-6}$ cells $h^{-1}$) Sensitive* | *Resistant* | *Fold increase in TS activity* | *TS protein level (pmol $10^{-6}$ cells) Sensitive* | *Resistant* | *Fold increase in TS protein* |
|---|---|---|---|---|---|---|
| CH1 (human ovarian) | 1.5 ± 0.66 | 6.3 ± 1.3 | 4.2* | 0.24 | 0.51 | 2 |
| | | | | 0.089 | 0.19 | 2 |
| 41M (human ovarian) | 2.0 ± 0.64 | 2.5 ± 0.49 | 1.3** | 0.15 | 0.092 | 0.6 |
| | | | | 0.22 | 0.11 | 0.5 |
| W1L2 (human lymphoblastoid) | 1.6 ± 0.33 | 820 ± 200 | 514* | 0.052, 0.012 | 5.7 | 178 |
| | | | | 0.089 | 16 | 180 |
| L1210 (murine leukaemia) | 1.4 ± 0.21 | 2.3 ± 0.91 | 1.6** | 0.022, 0.023 | 0.014 | 0.5 |
| | | | | 0.040 | 0.08 | 2 |

TS activity was measured by $^3H$ release from [5-$^3H$]dUMP (mean ± s.d. of at least three experiments: *$P<0.05$; **not significant) and TS protein levels by ELISA.

**Figure 1** Deoxypyrimidinenucleotide pool measurements. After extraction of the deoxynucleotides, TTP was measured by RIA using a specific antibody, and immunoreactive deoxyuridine nucleotides (mainly dUMP) were measured by RIA using a specific dUTP antibody that cross-reacts with dUMP and dUDP. TTP and 'dUMP' levels in controls were ~20 and 5 pmol $10^{-6}$ cells for the L1210 and W1L2 cells and ~80 and 10 pmol $10^{-6}$ cells for the CH1 and 41M cells respectively.

**Figure 2** The uptake of 0.1 μM [5-$^3$H]ZD1694 into parental (solid lines) and resistant (dashed lines) mouse L1210 (circles) and human ovarian 41M cells (squares). Cells were incubated in unsupplemented RPMI-1640 (L1210) or Dulbecco's modified Eagle medium (41M) at 37°C.

**Table III** Kinetics for the transport of methotrexate into L1210 and L1210:R$^{D1694}$

| | $K_t$ (μM) | $V_{max}$ (pmol $10^{-6}$ cells $min^{-1}$) | $K_i$ (μM) D1694 |
|---|---|---|---|
| L1210 | 4.2 ± 0.3 | 1.6 ± 0.07 | 2.4 ± 0.1 |
| | 4.0 ± 0.2 | 0.93 ± 0.14 | 2.6 ± 0.2 |
| L1210:R$^{D1694}$ | 3.8 ± 0.2 | 0.46 ± 0.02 | 3.7 ± 0.2 |
| | 3.5 ± 0.3 | 0.47 ± 0.02 | 3.6 ± 0.2 |

Cells were incubated in transport buffer with 0.5 μM [$^3$H]MTX for 5 min in the presence of varying concentrations of MTX or ZD1694. Each result (± s.e.) represents one experiment.

1.7 μM) compared with 60% and 80% reduction in TTP and 31- and 38-fold increase in dUMP in the parental line at these doses. Pool size perturbations in the CH1 and CH1: R$^{D1694}$ lines were not so markedly different. ZD1694 at 0.1 μM for 4 h caused TTP pools to fall by 81% and 58% in the sensitive and resistant line respectively and by 88% in both lines following 1 μM ZD1694. $IC_{50}$ values for growth inhibition of the resistant CH1 line was 0.35 μM ZD1694. 'dUMP' pools mirrored the changes in TTP pools.

### *Folylpolyglutamate synthetase activity*

FPGS activity was determined in the L1210 cell lines using D1694 as the substrate (3 μM). The activity in L1210 cells was 2.0 ± 0.84 pmol $h^{-1}$ $10^{-6}$, whereas for the L1210:R$^{D1694}$ cells it was only 0.25 ± 0.20 pmol $h^{-1}$ $10^{-6}$ cells. A similar difference in activity was observed using MTX as the substrate (data not shown).

### *Cellular transport/uptake studies*

The 41M and L1210 sensitive and resistant lines were incubated with 0.1 μM ZD1694 and the cellular levels measured up to 20 or 30 min (Figure 2). Uptake of the drug was significantly lower in both resistant lines compared with the parental cells (~15–25%). Closer examination of the L1210 parental cells revealed that within 15 min approximately half of the intracellular drug was present as polyglutamates (data not shown). [$^3$H]MTX transport was therefore measured in these cells, the advantage being that polyglutamate formation is relatively slow and no polyglutamates could be detected by 15 min (data not shown). There was no significant change in the affinity ($K_t$) of MTX for the RFC, but at least a 50% reduction in the $V_{max}$ was observed (Table III). The $K_i$ for ZD1694 as an inhibitor of MTX transport was only marginally higher for the resistant L1210 cells. This small alteration in the RFC is not sufficient to explain the > 11 000-fold resistance to ZD1694. Furthermore, very little cross-resistance was seen to MTX, suggesting that impaired transport is not a major determinant of resistance (see Table VIII). However, the rate of transport of [$^3$H]MTX into the 41M:R$^{D1694}$ cells was significantly reduced. At an extracellular concentration of 0.5 μM ~0.6 pmol of MTX had accumulated within 30 min in the 41M cells. This was only 0.2 pmol in the 41M:R$^{D1694}$ cells (data not shown). In addition, there was a concomitant 36-fold cross-resistance to MTX (123-fold resistant to ZD1694) (see Table VII). Further characterisation of the transport properties of these 41M cells was impaired by methodological problems with this adherent cell line. Reproducible data could not be obtained at short incubation times necessary to perform such experiments. Changing the method to using single-cell suspensions was not possible with this cell line as 'clumping' occurred during the incubation.

### *Polyglutamation of ZD1694, MTX and ICI 198583*

The total intracellular level of the drug in the 41M cells 24 h after exposure to 0.1 μM [$^3$H]ZD1694 was 8.8 μM, i.e. 23-fold higher than in the resistant 41M cells (Figure 3a). The levels of the parent monoglutamate and its polyglutamate forms were all reduced in the resistant cell line. However, the predominant metabolite was the triglutamate in both cell lines. These data are consistent with a reduced rate of membrane transport of ZD1694 in the resistant cells. The intracellular pool of ZD1694 (particularly the triglutamate) could be increased by raising the extracellular concentration to 1 μM. There was relatively more parent drug in the 41M:R$^{D1694}$ cells, which may indicate some reduction in the rate of polyglutamate formation. The uptake of 0.1 μM [$^3$H]ZD1694

over 24 h into L1210 cells resulted in a 38-fold higher intracellular concentration of the drug, 98% of which was present as polyglutamate forms (Figure 3b). However, in L1210: $R^{D1694}$ cells, the level was approximately equivalent to the extracellular concentration and no polyglutamates were found. Raising the extracellular level to 1 μM resulted in small, but detectable levels of polyglutamates. Resistance to ZD1694 is clearly associated with a severely reduced ability to form or maintain polyglutamates. To determine whether this was a general effect on antifolates, [$^3$H]MTX (0.5 μM) polyglutamation in the sensitive and resistant L1210 cell lines was compared. Relative to ZD1694, MTX polyglutamation was low in L1210 cells, with only ~20% of total (5.3 μM) intracellular drug being found as polyglutamates at 24 h (di-pentaglu). However, only parent drug (4.2 μM) was seen in the L1210:$R^{D1694}$ cells at the same dose (data not shown). [$^3$H]ICI 198583 (1 μM) was well metabolised to polyglutamates in L1210 cells (total intracellular drug at 24 h = ~3μM; 75% as di-pentaglutamates, principally tetraglutamate) (Jackman *et al.*, 1991*c*). Again the L1210:$R^{D1694}$ cells were unable to form polyglutamates, with 98% of the cellular $^3$H-labelled material (total = 2.6 μM) being identified as parent drug (data not shown).

After 24 h exposure to 0.1 μM [$^3$H]ZD1694, significant intracellular accumulation was found in W1L2:$R^{D1694}$ cells (~3 × W1L2 parental line). Most was parent drug (28%) or diglutamate (62%) (Figure 3c). In parental W1L2 cells only 1% and 2% of the drug was in these forms, the vast majority being tetra- and pentaglutamates.

The TS-overproducing cell line, CH1:$R^{D1694}$, did not accumulate a significantly higher level of ZD1694 when compared with parental cells. However, there was, at 4 and 24 h, a shift to a greater proportion in the mono- to triglutamate form in the resistant cells. The significance of this result is difficult to interpret (Figure 3d).

### *Cross-resistance studies*

The structures of the quinazoline TS inhibitors, together with their TS inhibitory activity and FPGS substrate activity, are given in Table IV. Results indicating their use of the RFC is indicated in Tables V–VIII, and details can be found in the following publications: Jackman *et al.* (1991*a*,*c*,*d*, 1993*a*,*b*), Marsham *et al.* (1991), Boyle *et al.* (1993). Details of the other compounds are described by Duch *et al.* (1993), Pavlovic (1993) and Webber *et al.* (1993).

W1L2:$R^{D1694}$ cells were highly cross-resistant to all quinazoline, pyrrolopyrimidine (LY231514) and benzoquinazoline (BW1843U89) TS inhibitors, consistent with their high expression of TS (Table V). Lower cross-resistance was seen to the lipophilic analogue, 10, which possibly relates to a second locus of action becoming important to growth inhibition at the higher compound concentration. No cross-resistance was

**Figure 3** Intracellular ZD1694 polyglutamate formation in parental and resistant cells. Cells were incubated for 4 or 24 h with 0.1 or 1 μM [5-$^3$H]ZD1694. After extraction of ZD1694 and its polyglutamates, analysis was performed using an ion-pairing HPLC system. Fractions were identified by the addition of the appropriate polyglutamate standards and radioactivity measured by liquid scintillation counting. ▭, Parent drug; ■, diglu; ▨, triglu; ▧, tetraglu; ▩, pentaglu; ▥, hexaglu.

**Table IV** Structures of quinazoline TS inhibitors

| | $R_1$ | $R_2$ | $R_3$ | *Aryl* | $R_4$ | *Inhibition of L1210 TS ($IC_{50}$, μM)* | *Mouse liver FPGS ($K_m$, μM)* |
|---|---|---|---|---|---|---|---|
| **1** (ZD1694) | $CH_3$ | H | $CH_3$ | Thiophene | Glu | 0.67 ($K_i$ = 60 nM) | [a]1.3 |
| **2** | $CH_3$ | $CH_3$ | $CH_3$ | Thiophene | Glu | 0.11 | [b]143 |
| **3** | $CH_3$ | H | $CH_3$ | Thiazole | Glu | 0.42 | [a]0.91 |
| **4** (ICI 198583) | $CH_3$ | H | $CH_2CCH$ | Benzene | Glu | 0.05 ($K_i$ = 10 nM) | [a]43 |
| **5** (CB3717) | $NH_2$ | H | $CH_2CCH$ | Benzene | Glu | 0.02 ($K_i$ = 3 nM) | [a]40 |
| **6** | $CH_3$ | $CH_3$ | $CH_2CCH$ | Benzene | Glu | 0.028 | [b]Non-sub |
| **7** | $CH_3$ | H | H | Benzene | Glu | 4.5 | [a]6.4 |
| **8** | $CH_3$ | H | $CH_2CCH$ | Benzene | Val | 0.06 | Non-sub |
| **9** | $CH_3$ | $CH_3$ | $CH_2CCH$ | 2'F benzene | L-Glu-γ-D-Glu | 0.00092 ($K_i$ = 0.2 nM) | Non-sub |
| **10** | $CH_3$ | $CH_3$ | $CH_2CCH$ | Benzene | *meta*-CN benzylamide | 0.026 | Non-sub |

[a]Jackman *et al.* (1991*d*); [b]Sanghani *et al.* (1994).

**Table V** Activity of antifolates against W1L2 and W1L2:$R^{ICI\ D1694}$ human lymphoblastoid cells

| | *Locus* | [a]*RFC* | *FPGS* | *Inhibition of W1L2 cell growth* S ($IC_{50}$, μM) | $R^{D1694}$ | *Resistance factor R/S* |
|---|---|---|---|---|---|---|
| **1** (ICI D1694) | TS | + | + | 0.0046 ± 0.001 | >100 (*n* = 2) | >22 000* |
| **2** | TS | + | +/− | 0.70, 0.76 | >100 | >140 |
| **3** | TS | + | + | 0.0029, 0.011 | >100 | 14 000 |
| **4** (ICI 198583) | TS | + | + | 0.058 ± 0.0074 | >100 (*n* = 2) | >1700* |
| **5** (CB3717) | TS | − | + | 2.6 ± 0.29 | >100 (*n* = 2) | >38* |
| **6** | TS | + | − | 0.15 ± 0.047 | >100 (*n* = 2) | >830* |
| **7** | TS | + | + | 0.031 ± 0.0023 | >100 | >3000 |
| **8** | TS | + | − | 0.42, 0.35 | >100 | >260 |
| **9** | TS | + | − | 0.17, 0.093 | 63 | 630 |
| **10** | TS | − | − | 0.068, 0.084 | 11, 18 | 190 |
| LY231514 | TS | + | + | 0.029, 0.056 | >100 (*n* = 2) | >2400 |
| BW1843U89 | TS | + | + | 0.0023, 0.0022 | 3.4, 4.2 | 1700 |
| AG337 | TS | − | − | 0.78 ± 0.04 | >10 | >58 |
| FdUrd | TS | − | − | 0.0054 ± 0.0013 | 0.0078 ± 0.0069 | 1.4** |
| FdUrd + 5 μM LV | TS | − | + | 0.0015 | 0.95 | 630 |
| Trimetrexate | DHFR | − | − | 0.01, 0.018 | 0.023 ± 0.021 | 1.3** |
| Methotrexate | DHFR | + | + | 0.0094 ± 0.0018 | 0.0026, 0.0021 | 0.26* |
| Lometrexol | GARTF | + | + | 0.033 ± 0.011 | 0.074, 0.045 | 1.8 |

[a]Determined by inhibition of [$^3$H]MTX transport and/or activity against resistant cell lines with an impaired RFC (Jackman *et al.*, 1991*a,c,d*, 1993*a,b*; Marsham *et al.*, 1991; Boyle *et al.*, 1993; Duch *et al.*, 1993; Pavlovic *et al.*, 1993; Webber *et al.*, 1993). The significance of the difference between $IC_{50}$ values for the S and R cells is: **P*<0.05; **Not significant.

**Table VI** Activity of antifolates against human ovarian CH1 and CH1:$R^{D1695}$ cells

| *Compound* | *Locus* | *RFC* | *FPGS* | *Inhibition of CH1 cell growth* S ($IC_{50}$, μM) | $R^{D1694}$ | *Resistance factor R/S* |
|---|---|---|---|---|---|---|
| **1** (ICI D1694) | TS | + | + | 0.025 ± 0.011 | 0.36 ± 0.12 | 14* |
| **4** (ICI 198583) | TS | + | + | 0.34 ± 0.17 | 4.7, 7.0 | 17* |
| **5** (CB3717) | TS | − | + | 7.0 ± 1.8 | 55, 91 | 10* |
| **6** | TS | + | − | 7.9 ± 4.7 | 167 ± 97 | 21* |
| **8** | TS | + | − | 41 | ~250 | ~7 |
| **9** | TS | + | − | 1.3 ± 0.26 | 9.5 ± 3.9 | 7.3* |
| LY231514 | TS | + | + | 3.0 ± 3.0 | 67 ± 2.9 | 22* |
| BW1843U89 | TS | + | + | 0.0012 ± 0.0006 | 0.0084 ± 0.0025 | 7* |
| AG337 | TS | − | − | 9.3 ± 1.2 | 22 ± 6.2 | 2.4* |
| FdUrd | TS | − | − | 0.044 ± 0.025 | 0.41 ± 0.23 | 9.3* |
| Trimetrexate | DHFR | − | − | 0.0086, 0.062 | 0.0073, 0.094 | 1.5 |
| Methotrexate | DHFR | + | + | 0.017, 0.019 | 0.018, 0.0043 | 1.7 |
| Lometrexol | GARTF | + | + | 0.42 ± 0.055 | 0.48 ± 0.2 | 1.1** |

**P*<0.05; **Not significant.

Table VII Activity of antifolates against human ovarian 41M and 41M:R[D1694] cells

| | Locus | RFC | FPGS | Inhibition of 41M cell growth S ($IC_{50}$, μM) | $R^{D1694}$ | Resistance factor R/S |
|---|---|---|---|---|---|---|
| 1 (ICI D1694) | TS | + | + | 0.013 ± 0.0043 | 1.7 ± 0.63 | 123* |
| 4 (ICI 198583) | TS | + | + | 0.13 ± 0.06 | 5.3 ± 2.9 | 41* |
| 5 (CB3717) | TS | − | + | 5.6 ± 2.7 | 7.6 ± 4.5 | 1.4** |
| 6 | TS | + | − | 21 ± 15 | 131 ± 56 | 9.9* |
| 7 | TS | + | + | 0.19 ± 0.02 | 8.8 ± 4.9 | 46* |
| 8 | TS | + | − | >50, ~140 | >100 | - |
| 9 | TS | + | − | 3.3 ± 2.1 | 20 ± 4.2 | 6.1* |
| LY231514 | TS | + | + | 0.22, 0.22 | 1.4, 2.0 | 7.7 |
| BW1843U89 | TS | + | + | 0.0025 ± 0.0007 | 0.66 ± 0.065 | 264* |
| AG337 | TS | − | − | 6.7 ± 2.7 | 6.5 ± 3.1 | 0.97** |
| FdUrd | TS | − | − | 0.02 ± 0.006 | 0.042 ± 0.02 | 2.1** |
| Trimetrexate | DHFR | − | − | 0.082 ± 0.068 | 0.015, 0.068 | 0.45** |
| Methotrexate | DHFR | + | + | 0.025 ± 0.015 | 0.69, 1.1 | 36 |
| Lomotrexol | GARTF | + | + | 2.8 ± 1.4 | 107, 100 | 37* |

*$P < 0.05$; **Not significant.

Table VIII Activity of antifolates against mouse L1210 and L1210:R[D1694] cells

| Compound | Locus | ᵃRFC | FPGS | Inhibition of L1210 cell growth ($IC_{50}$, μM) S | $R^{D1694}$ (MB3) | Resistance factor R/S |
|---|---|---|---|---|---|---|
| 1 (ICI D1694) | TS | + | + | 0.0088 ± 0.0031 | >100 n = 3 | >11 000* |
| 2 | TS | + | +/− | 1.6 ± 0.3 | 9, 10 | 5.9* |
| 3 | TS | + | + | 0.0060 ± 0.0022 | 18, 15 | 3 000* |
| 4 (ICI 198583) | TS | + | + | 0.15 ± 0.036 | 2.7 ± 0.47 | 18* |
| 5 (CB3717) | TS | − | + | 5.0 ± 1.2 | 13 ± 2.1 | 2.6* |
| 6 | TS | + | − | 0.21 ± 0.036 | 0.56, 0.62 | 2.8* |
| 7 | TS | + | + | 0.072 ± 0.002 | >100 n = 2 | >1400* |
| 8 | TS | + | − | 1.1 ± 0.51 | 3.7 ± 1.1 | 3.4* |
| 9 | TS | + | − | 0.2 ± 0.017 | 0.3, 0.52 | 2 |
| 10 | TS | − | − | 0.89 ± 0.19 | 0.35 ± 0.18 | 0.4* |
| LY231514 | TS | + | + | 0.034 ± 0.006 | 3.9, 5.1 | 132 |
| BW1843U89 | TS | + | + | 0.086 ± 0.045 | 0.36, 0.43 | 4.7* |
| AG337 | TS | − | − | 0.70 ± 0.04 | 1.4, 1.2 | 1.8 |
| FdUrd | TS | − | − | 0.0023 ± 0.001 | 0.0035 ± 0.0016 | 1.5** |
| Trimetrexate | DHFR | − | − | 0.018 ± 0.0076 | 0.016 ± 0.0087 | 0.89** |
| Methotrexate | DHFR | + | + | 0.011 ± 0.0035 | 0.024 ± 0.003 | 2.2* |
| Aminopterin | DHFR | + | + | 0.0024, 0.0024 | 0.0075, 0.004 | 2.4 |
| Lomotrexol | GARTF | + | + | 0.11 ± 0.041 | 2.3 ± 0.41 | 21* |

*$P < 0.05$; **Not significant.

observed to the pyrimidine-based TS inhibitor 5-fluorodeoxyuridine (FdUrd) unless folinic acid (leucovorin, LV) was given. The addition of dThd (as well as LV) rendered FdUrd poorly active in either cell line ($IC_{50}$ ~65 μM). Inhibitors of DHFR (MTX and trimetrexate) and glycinamide ribotide transformylase (GARTF) (Lometrexol) retained activity in the ZD1694-resistant W1L2 cells.

Lower levels of resistance to ZD1694 and other TS inhibitors, including FdUrd, were seen in the CH1:R[D1694] cells (2- to 22-fold) (Table VI). The 2.4 cross-resistance to AG337 may relate to its poor activity against the parental line (9.3 μM) with the possibility of another locus becoming significant at the higher concentration used against the resistant cells. No significant cross-resistance was seen to inhibitors of other folate-dependent enzymes, e.g. MTX, trimetrexate or Lometrexol.

The pattern of cross-resistance to the antifolates was very different in the other two cell lines, and the results are crucial in explaining the mechanisms of resistance involved. This is because the data separating transport from an FPGS defect were not definitive (see above). The 41M:R [D1694] cells were only cross-resistant to compounds (whatever the target enzyme) known to use the RFC, although the degree of cross-resistance varied widely (6- to 264-fold) (Table VII).

The L1210:R[D1694] cell line produced cross-resistance results that can all be explained as a consequence of defective polyglutamation (Table VIII). High cross-resistance was observed to the thiazole derivative of ZD1694 (**3**) and to **7**, both very good FPGS substrates ($K_m$ = 1.0 and 6.4 μM respectively) (Jackman *et al.*, 1991*d*). The degree of cross-resistance to other analogues was lower, and was again consistent with their reduced potential to form intracellular polyglutamates. Thus, ICI 198583 with a relatively high $K_m$ for FPGS (40 μM) is less extensively polyglutamated in L1210 cells than ZD1694 (Jackman *et al.*, 1991*c*). No significant cross-resistance was seen to all antifolates known to be non-substrates for FPGS and that do not use the RFC, i.e. **10**, AG337 and trimetrexate. Similarly no cross-resistance was observed to FdUrd. Non-polyglutamatable compounds that use the RFC (e.g. **6**, **8** and **9** and other unpublished examples) have cross-resistance values (2–3) significantly greater than 1, consistent with the small transport defect in the resistant line described above. A very low level of cross-resistance was observed to CB3717, MTX and aminopterin (2- to 3-fold). However a higher level of resistance (15-fold) was demonstrated to MTX if the drug exposure period was limited to 6 h (data not shown). Indirect evidence supports polyglutamation of natural folates occurring normally in the L1210:R[D1694] cells. The addition of 5 μM leucovorin to cell cultures containing FdUrd resulted in a significant enhancement in growth inhibition (~2.5-fold) in both lines (data not shown). This is a well-recognised synergistic combination since stable ternary complex formation is promoted by elevating the 5,10-$CH_2FH_4$ pool with leucovorin. Similarly, when leucovorin (5 μM) was used to protect against the activity of the DHFR inhibitor, trimetrexate, both sensitive and resistant L1210 cells were protected to the same degree (~20-fold increase in $IC_{50}$). Since leucovorin is metabolised to natural folate polyglutamates, this provides circumstantial evidence for normal folate polyglutamation in L1210:R[D1694] cells.

**Discussion**

Continuous exposure of four cell lines to incremental increases in the concentration of ZD1694 led to each line acquiring at least one of three mechanisms of resistance: TS overproduction, alteration in the RFC or defective polyglutamation. There was always one primary mechanism of resistance, although a second mechanism (either independent or a consequence of the first) was also observed in each cell line.

Although the cell lines were adapted to grow in 1–5 $\mu$M ZD1694, two of the resistant cell lines (W1L2 and L1210) were highly resistant, even when challenged with 100 $\mu$M ZD1694. Consistent with TS inhibition, all parental cells had depletion of TTP and elevation of 'dUMP' pools following incubation for 4 h with growth-inhibitory concentrations of ZD1694. These pools were either not, or minimally, disturbed when L1210 or W1L2 resistant cells were exposed to 50 $\mu$M ZD1694, demonstrating that TS operates relatively normally under these conditions. This would be expected from the high level of resistance described above. The two resistant ovarian lines, particularly the CH1:$R^{D1694}$ line, showed disturbances in pool size at much lower concentrations consistent with their lower levels of resistance.

Two of the human cell lines, W1L2 and CH1, had significantly elevated TS activity (500- and 4-fold respectively). TS protein levels were similarly increased when determined by ELISA (Table I). Immunoblotting demonstrated a similar increase, and this was accompanied by a 100- and 2- to 3-fold increase in TS gene copy number and mRNA respectively (Freemantle *et al.*, 1993). The human ovarian cell line, CH1, has a low level (14-fold) of ZD1694 resistance, and here the resistance is generally consistent with the small elevation in TS activity (levels). No change in the TS inhibition kinetic parameters (ZD1694 or its triglutamate) was observed. As expected, cross-resistance was seen to all TS inhibitors, including FdUrd, but not to inhibitors of DHFR (MTX or trimetrexate) or GARTF (Lometrexol). Mechanism of cellular uptake was not a factor that correlated with cross-resistance, and indeed intracellular ZD1694 levels were not significantly different in the sensitive and resistant cells. However at 24 h, a consistent shift in the distribution of the polyglutamate forms to shorter chain length was apparent in the resistant line, which may contribute to the resistance seen.

The human W1L2 resistant cell line was highly resistant to ZD1694 ($IC_{50} > 100\ \mu M$) even though it had been adapted to grow in just 5 $\mu$M drug. Contrary to what we expected, the resistant cells accumulated significantly higher levels of intracellular drug than the sensitive cells. However, the majority was present as either the parent drug or diglutamate, and this is most readily explained by the 500-fold increased level of TS protein. The high level of TS may reduce 'free' drug levels by binding the mono- to triglutamate, preventing the addition of further glutamate residues by FPGS. The relatively weaker binding to TS of the mono- and diglutamate compared with the higher polyglutamates must allow polyglutamation to proceed as far as the triglutamate, but the slower dissociation of this form from TS may prevent further polyglutamate chain elongation by reducing substrate availability. This phenomenon has also been observed in another TS-overproducing line (W1L2:C1) with resistance to ICI 198583. Here there was a 30-fold higher intracellular concentration of drug with 97% in the monoglutamate form compared with 9% in the parental cells (O'Connor *et al.*, 1992). The lower level of cross-resistance to quinazoline TS inhibitors that are not substrates for FPGS, e.g. **9** and **10**, is therefore expected. Sensitivity to the DHFR inhibitors (MTX and trimetrexate) and the GARTF inhibitor (Lometrexol) is completely consistent with TS overproduction being the only alteration in the W1L2:$R^{D1694}$ cells. Sensitivity to FdUrd is explained by sequestration of the 5,10-$CH_2FH_4$ pool into the ternary complex with TS (in large excess) and FdUMP, resulting in inhibition of TS and other folate-dependent enzymes through substrate depletion. This is why supplying the cell with folinic acid, which reinstates *de novo* purine and TMP synthesis by replenishment of the reduced folate pool, allows resistance to FdUrd to be revealed. We have previously recorded this phenomenon in other cell lines with amplified TS genes (Jackman *et al.*, 1986; O'Connor *et al.*, 1992).

Gene amplification leading to increased TS expression as a mechanism of experimental resistance to pyrimidine-based (FdUrd) (Baskin *et al.*, 1975; Priest *et al.*, 1980; Rossana *et al.*, 1982; Berger *et al.*, 1985; Jenh *et al.*, 1985) or folate-based TS inhibitors has been previously reported (Jackman *et al.*, 1986; Iman *et al.*, 1987; Danenberg and Danenberg, 1989; O'Connor *et al.*, 1992). There is also evidence that this can occur in man in response to treatment with 5-FU (Clark *et al.*, 1987). The introduction of new antifolates, targeted towards TS, into the clinic may establish whether this will be a common resistance mechanism in man.

The second human ovarian 41M line (~100-fold resistant to ZD1694) appears to have a primary defect, which is reduced drug uptake via the RFC. A consequence of lower intracellular level of the parent drug is a substantially reduced concentration of the more active intracellular polyglutamates. Thus, the cross-resistance seen to antifolates known to use the RFC, including those with targets other than TS, was expected. CB3717 uses this carrier poorly (as determined in L1210 cells) (Jackman *et al.*, 1990, 1993*a*), while the lipophilic compounds AG337 (Webber *et al.*, 1993) and trimetrexate (Fry and Jackson, 1986) do not use it at all, and of course FdUrd uses a nucleoside transport mechanism. All of these compounds retained activity in the resistant cells. The very high cross-resistance seen to BW1843U89 may relate to the excellent affinity this compound has for the human RFC (Duch *et al.*, 1993). Transport defects have frequently been reported as a mechanism of resistance to MTX (Fry and Jackson, 1986). Defective transport is also commonly associated with other resistance mechanisms, including a reduced ability to form or maintain polyglutamates of MTX or Lometrexol (Cowan and Jolivet, 1984; Assaraf *et al.*, 1992; Koizumi and Allegra, 1992; Rhee *et al.*, 1993).

The importance of polyglutamation in the action of antifolates such as MTX and Lometrexol has led to the recent description of several cell lines with intrinsic or acquired resistance due to a reduced accumulation of polyglutamate species (Pizzorno *et al.*, 1988, 1989; McCloskey *et al.*, 1991; Van der Laan *et al.*, 1991; Li *et al.*, 1993; Pavlovic *et al.*, 1993). Considering the importance of polyglutamate formation for the cytotoxicity of ZD1694 (Jackman *et al.*, 1991*a*, 1993*a*), it is not surprising that one of our cell lines, L1210:$R^{D1694}$, proved to be defective in this manner. The greatly reduced ability to form (or maintain) intracellular ZD1694 polyglutamates in this resistant line also extends to ICI 198583 and MTX as determined by radiolabelled drug studies. This reduction in polyglutamation potential is most likely accounted for by the ~90% reduction in FPGS activity (using MTX or ZD1694 as substrates). Deletion of FPGS has been shown to be a lethal mutation (McBurney and Whitmore, 1974), but reports of lower expression of FPGS or altered activity for antifolates (MTX and Lometrexol) has been described (Pizzorno *et al.*, 1988; McCloskey *et al.*, 1991; Pavlovic *et al.*, 1993). It has also been shown that decreased FPGS activity does not necessarily reduce the ability of the cell to polyglutamate sufficient natural folates for normal cellular function (Pizzorno *et al.*, 1988; Pavlovic *et al.*, 1993). In other cell lines with decreased ability to polyglutamate MTX the actual mechanisms have not been fully elucidated (Pizzorno *et al.*, 1988, 1989; Assaraf *et al.*, 1992). Increased $\gamma$-glutamylhydrolase activity as a mechanism of antifolate resistance is increasingly being reported (Li *et al.*, 1993; Rhee *et al.*, 1993). However, in studies not reported here, this activity was barely detectable in both sensitive and resistant L1210 cells and is therefore unlikely to be a contributory factor. The very low activity of $\gamma$-glutamylhydrolase in L1210 cells grown *in vitro*, as opposed to in mice, has been described by Samuels *et al.* (1986). Thus, this enzyme may play a greater role in maintaining polyglutamate homeostasis in mice and may be important in differential tissue sensitivity to ZD1694, and its elevation could be a potential resistance mechanism.

The results of the cross-resistance studies support a polyglutamation defect, as only TS inhibitors, active through polyglutamation, had significantly reduced activity against the L1210 resistant cells. Some apparent anomalies do exist but can be explained including the low level of cross-resistance to CB3717 (3-fold) or to the pteridine-based DHFR inhibitors, MTX and aminopterin (2- to 3-fold). CB3717 does form polyglutamates in L1210 cells, but relatively slowly. For example, after incubation with equitoxic concentrations of ZD1694 (0.1 μM) and CB3717 (50 μM) for 4 or 6 h respectively, 98% of D1694 and only 15% of CB3717 was measured as polyglutamates (Sikora *et al*., 1988; Jackman *et al*., 1991*a*). CB3717's lower dependence on polyglutamate formation for activity, in addition to the slightly lower cross-resistance expected from a compound that does not use the RFC (small transport defect, see above), probably explains the low cross-resistance observed. A lower requirement for polyglutamation may partially explain the good activity of MTX and aminopterin in the resistant cells (MTX is poorly polyglutamated at low intracellular concentrations). Furthermore, the polyglutamates of DHFR inhibitors, unlike the quinazoline TS inhibitors, are not significantly more potent as inhibitors of their target enzyme (Szeto *et al*., 1979; Chabner *et al*., 1985) than the parent monoglutamate, and the importance of their formation lies in their drug retentive properties (Galivan, 1980; Jolivet and Chabner, 1983). Thus, under continuous drug exposure conditions, the lack of polyglutamate formation is not particularly deleterious, however under short exposure conditions (6 h), when drug retention is important, MTX was ~15-fold less active in the resistant than in the L1210 parental line. Similarly, the TS inhibitor BW1843U89 has similar activity against TS in either the parent monoglutamate or diglutamate form (Duch *et al*., 1993) and may therefore account for the low level of cross-resistance to this agent.

The acquisition of these four ZD1694-resistant cell lines has confirmed the importance of TS, the RFC and polyglutamation for the activity of the drug. Clearly, it is likely that certain tumours may intrinsically possess or acquire these phenotypes. However, as more antifolates are developed for clinical study with differing target enzymes, transport and metabolic properties, it will be possible to overcome these resistance mechanisms. This panel of resistant cell lines has aided the development of quinazoline TS inhibitors that are not metabolised by FPGS but still require cellular uptake via the RFC and are currently being used to identify compounds whose activity is independent of either process.

**Acknowledgements**
This work was supported by the Cancer Research Campaign, UK. The authors are grateful to Dr W Ward of Zeneca Pharmaceuticals who performed the data analysis for the TS inhibition kinetics.

**References**

AHERNE GW, HARDCASTLE A AND NEWTON R. (1992). Measurement of human thymidylate synthase (hTS) in cell lines using ELISA. *Ann. Oncol*, 3 (Suppl. 5), 77.

AHERNE W, HARDCASTLE A AND JACKMAN AL. (1993). Radioimmunoassay of dTTP pools in L1210 cells following thymidylate synthase inhibition. *Proc. Am. Assoc. Cancer Res.*, **34**, 273.

ASSARAF YG, FEDER JN, SHARMA RC, WRIGHT JE, ROSOWSKY A, SHANE B AND SCHIMKE RT. (1992). Characterisation of the coexisting multiple mechanisms of methotrexate resistance in mouse 3T6 R50 fibroblasts. *J. Biol. Chem.*, **267**, 5776-5784.

BARKER AJ, BOYLE FT, JACKMAN AL, CALVERT AH, PEGG SJ AND HUGHES LR. (1991). *In vitro* activity of non-glutamate containing quinazoline-based thymidylate synthase inhibitors. *Proc. Am. Assoc. Cancer Res.*, **32**, 327.

BASKIN F, CARLIN SC, KRAUS P, FRIEDKIN M AND ROSENBERG RN. (1975). Experimental chemotherapy of neuroblastoma. *Mol. Pharmacol.*, **11**, 105-117.

BAVETSIAS V, JACKMAN AL, THORNTON TJ, PAWELCZAK K, BOYLE FT AND BISSET GMF. (1993). Quinazoline antifolates inhibiting thymidylate synthase: synthesis of γ-linked peptide and amide analogues of 2-desamino-2-methyl-$N^{10}$-propargyl-5,8-dideazafolic acid (ICI 198583). In *Advances in Experimental Medicine and Biology*, Vol. 338, Ayling JE, Nair MG and Baugh CM (eds) pp. 593-596. Plenum Press: New York.

BERGER, SH, JENH C-H, JOHNSON LF AND BERGER FG. (1985). Thymidylate synthase overproduction and gene amplification in fluorodeoxyuridine-resistant human cells. *Mol. Pharmacol.*, **28**, 461-467.

BOYLE FT, MATUSIAK ZS, HUGHES LR, SLATER AM, STEPHENS TC, SMITH MN, KIMBELL R AND JACKMAN AL. (1993). Substituted-2-desamino-2-methyl-quinazolinones. A series of novel antitumour agents. In *Advances in Experimental Medicine and Biology*, Vol. 338, Ayling JE, Nair MG and Baugh CM (eds) pp. 585-588. Plenum Press: New York.

BURRIS III H, VON HOFF D, BOERN K, HEAVEN R, RINALDI D, ECKARDT J, FIELDS S, CAMPBELL L, ROBERT F, PATTON S AND KENNEALEY G. (1994). A phase II trial of ZD1694, a novel thymidylate synthase inhibitor, in patients with advanced non-small cell lung cancer. *Ann. Oncol.*, **5** (Suppl. 5), 133.

CALVERT AH, ALISON DL, HARLAND SJ, ROBINSON BA, JACKMAN AL, JONES TR, NEWELL DR, SIDDIK ZH, WILTSHAW E, McELWAIN TJ, SMITH IE AND HARRAP KR. (1986). A phase I evaluation of the quinazoline antifolate thymidylate synthase inhibitor, $N^{10}$-propargyl-5,8-dideazafolic acid, CB3717. *J. Clin. Oncol.*, **4**, 1245-1252.

CHABNER BA, ALLEGRA CJ, CURT GA, CLENDENIN NJ, BARAM J, KOIZUMI S, DRAKE JC AND JOLIVET J. (1985). Polyglutamation of methotrexate. *J. Clin. Invest.*, **76**, 907-912.

CLARK JL, BERGER SH, MITTELMAN A AND BERGER FG. (1987). Thymidylate synthase gene amplification in a colon tumour resistant to fluoropyrimidine chemotherapy. *Cancer Treat. Rep.*, **71**, 261-265.

CLARKE SJ, JACKMAN AL AND JUDSON IR. (1993). The history of the development and clinical use of CB3717 and ICI D1694. In *Novel Approaches to Selective Treatments of Human Solid Tumours: Laboratory and Clinical Correlation*, Vol. 339, Rustum Y (ed.) pp. 277-287. Plenum Press: New York.

CLARKE SJ, WARD J, DE BOER M, PLANTING A, VERWEIJ J, SUTCLIFFE F, AZAB M AND JUDSON IR. (1994). Phase I study of the new thymidylate synthase inhibitor Tomudex (ZD1694) in patients with advanced malignancy. *Ann. Oncol.*, **5** (Suppl. 5), 132.

COWAN KH AND JOLIVET J. (1984). A methotrexate-resistant human breast cancer cell line with multiple defects, including diminished formation of methotrexate polyglutamates. *J. Biol. Chem.*, **259**, 10793-10800.

CURTIN NJ, HARRIS AL AND AHERNE GW. (1991). Mechanism of cell death following thymidylate synthase inhibition: 2'-deoxyuridine-5'-triphosphate accumulation, DNA damage, and growth inhibition following exposure to CB3717 and dipyridamole. *Cancer Res.*, **51**, 2346-2352.

DANENBERG KD AND DANENBERG PV. (1989). Activity of thymidylate synthetase and its inhibition by 5-fluorouracil in highly enzyme overproducing cells resistant to 10-propargyl-5,8-dideazafolate. *Mol. Pharmacol.*, **36**, 219-223.

DUCH DS, BANKS S, DEV IK, DICKERSON SH, FERONE R, HEATH LS, HUMPHREYS J, KNICK V, PENDERGAST W, SINGER S, SMITH GK, WATERS K AND WILSON HR. (1993). Biochemical and cellular pharmacology of 1843U89, a novel benzoquinazoline inhibitor of thymidylate synthase. *Cancer Res.*, **53**, 810-818.

FREEMANTLE SJ, LUNEC J, JACKMAN AL, KELLAND LR, AHERNE GW AND CALVERT AH. (1993). Molecular characterisation of three cell lines selected for resistance to folate-based thymidylate synthase inhibitors. *Br. J. Cancer*, **67** (Supp. XX), 79.

FRY DW AND JACKSON RC. (1986). Membrane transport alterations as a mechanism of resistance to anticancer agents. *Cancer Surveys*, **5**, 47-51.

FRY DW, BESSERER JA AND BORITZKI TJ. (1984). Transport of antitumour antibiotic CI-920 into L1210 leukemia cells by the reduced folate carrier system. *Cancer Res.*, **44**, 3366-3370.

GALIVAN J. (1980). Evidence for the cytotoxic activity of polyglutamate derivatives of methotrexate. *Mol. Pharmacol.*, **17**, 105-110.

GIBSON W, BISSET GMF, MARSHAM PR, KELLAND LR, JUDSON IR AND JACKMAN AL. (1993). The measurement of polyglutamate metabolites of the thymidylate synthase inhibitor, ICI D1694, in mouse and human cultured cells. *Biochem. Pharmacol.*, **45**, 863-869.

GRINDEY GB, SHIH C, BARNETT CJ, PEARCE HL, ENGELHARDT JA, TODD GC, RNZEL SM, WORZALLA JF, GOSSETT LS, EVERSON TP, WILSON TM, KOBIERSKI ME, WINTER MA, BEWLEY JR, KUHNT D, TAYLOR EC AND MORAN RG. (1992). LY231514, a novel pyrrolopyrimidine antifolate that inhibits thymidylate synthase. *Proc. Am. Assoc. Cancer Res.*, **33**, 441.

GORE M, EARL H, CASSIDY J, TATTERSAL M, MANSI J AND AZAB M. (1994). Phase II study of Tomudex (ZD1694) in refractory ovarian cancer. *Ann. Oncol.*, **5** (Suppl. 5), 133.

HILLS CA, KELLAND LR, ABEL G, SIRACKY J, WILSON AP AND HARRAP KR. (1989). Biological properties of ten human carcinoma cell lines: calibration *in vitro* against four platinum complexes. *Br. J. Cancer*, **59**, 527-534.

HUGHES LR, JACKMAN AL, OLDFIELD J, SMITH RC, BURROWS KD, MARSHAM PR, BISHOP JAM, JONES TR, O'CONNOR BM AND CALVERT AH. (1990). Quinazoline antifolate thymidylate synthase inhibitors: alkyl, substituted alkyl, and aryl substituents in the C2 position. *J. Med. Chem.*, **33**, 3060-3067.

IMAM AMA, CROSSLEY PH, JACKMAN AL AND LITTLE PFR. (1987). Analysis of thymidylate synthetase gene amplification and of mRNA levels in the cell cycle. *J. Biol. Chem.*, **262**, 7368- 7373.

JACKMAN AL, ALISON DL, CALVERT AH AND HARRAP KR. (1986). Increased thymidylate synthase in L1210 cells possessing acquired resistance to $N^{10}$-propargyl-5,8-dideazafolic acid (CB3717): development, chracterisation and cross-resistance studies. *Cancer Res.*, **46**, 2810-2815.

JACKMAN AL, TAYLOR GA, O'CONNOR BM, BISHOP JA, MORAN RG AND CALVERT AH. (1990). Activity of the thymidylate synthase inhibitor 2-desamino-$N^{10}$-propargyl-5,8-dideazafolic acid and related compounds in murine (L1210) and human (W1L2) systems *in vitro* and in L1210 *in vivo*. *Cancer Res.*, **50**, 5212-5218.

JACKMAN AL, TAYLOR GA, GIBSON W, KIMBELL R, BROWN M, CALVERT AH, JUDSON IR AND HUGHES LR. (1991*a*). ICI D1694, a quinazoline antifolate thymidylate synthase inhibitor that is a potent inhibitor of L1210 tumor cell growth *in vitro* and *in vivo*: a new agent for clinical study. *Cancer Res.*, **51**, 5579-5586.

JACKMAN AL, JODRELL DI, GIBSON W AND STEPHENS TC. (1991*b*). ICI D1694, an inhibitor of thymidylate synthase for clinical study. In *Purine and Pyrimidine Metabolism in Man*, Vol. VII, Part A. (RA Harkness and TB Elion, (eds) pp. 19-23. Plenum Press: New York.

JACKMAN AL, NEWELL DR, GIBSON W, JODRELL DI, TAYLOR GA, BISHOP JA, HUGHES LR AND CALVERT AH. (1991*c*). The biochemical pharmacology of the thymidylate synthase inhibitor, 2-desamino-2-methyl-$N^{10}$-propargyl-5,8-dideazafolic acid (ICI 198583). *Biochem. Pharmacol.*, **42**, 1885-1895.

JACKMAN AL, MARSHAM PR, MORAN RG, KIMBELL R, O'CONNOR BM, HUGHES LR AND CALVERT AH. (1991*d*). Thymidylate synthase inhibitors: the *in vitro* activity of a series of heterocyclic benzoyl ring modified 2-desamino-2-methyl-$N^{10}$-substituted-5,8-dideazafolates. *Adv. Enz. Regul.*, **31**, 13-27.

JACKMAN AL, GIBSON W, BROWN M, KIMBELL R AND BOYLE FT. (1993*a*). The role of the reduced-folate carrier and metabolism to intracellular polyglutamates for the activity of ICI D1694. In *Novel Approaches to selective Treatments of Human Solid Tumors: Laboratory and Clinical Correlation*, Vol. 339, Rustum Y (ed.) pp. 265-276. Plenum Press: New York.

JACKMAN AL, BISSET GMF, JODRELL DI, GIBSON W, KIMBELL R, BAVETSIAS V, CALVERT AH, HARRAP KR, STEPHENS TC, SMITH MN AND BOYLE FT. (1993*b*). $\gamma$-linked dipeptide analogues of 2-desamino-2-methyl-$N^{10}$-propargyl-5,8-dideazafolate as antitumour agents. In *Advances in Experimental Medicine and Biology*, Vol. 338, Ayling JE, Nair MG and Baugh CM. (eds) pp. 579-584. Plenum Press: New York.

JANSEN G, SCHORNAGEL JH, WESTERHOF GR, RIJKSON GR, NEWELL DR AND JACKMAN AL. (1990). Multiple membrane transport systems for the uptake of folate-based thymidylate synthase inhibitors. *Cancer Res.*, **50**, 7422-7548.

JENH C-H, GEYER PK, BASKIN F AND JOHNSON LF. (1985). Thymidylate synthase gene amplification in fluorodeoxyuridine-resistant mouse cell lines. *Mol. Pharmacol.*, **28**, 80-85.

JENNRICH RJ AND SAMPSON PF. (1968). Application of a step-wise regression to non-linear least squares estimation. *Technometrics*, **10**, 63-72.

JOLIVET J AND CHABNER BA. (1983). Intracellular pharmacokinetics of methotrexate polyglutamates in human breast cancer cells. *J. Clin. Invest.*, **72**, 773-778.

JONES TR, CALVERT AH, JACKMAN AL, BROWN SJ, JONES M AND HARRAP KR. (1981). A potent antitumour quinazoline inhibitor of thymidylate synthetase: synthesis, biological properties and therapeutic results in mice. *Eur. J. Cancer*, **17**, 11-19.

JONES TR, THORNTON TJ, FLINN A, JACKMAN AL, NEWELL DR AND CALVERT AH. (1989). Quinazoline antifolates inhibiting thymidylate synthase: 2-desamino derivatives with enhanced solubility and potency. *J. Med. Chem.*, **32**, 847-852.

KOIZUMI S AND ALLEGRA CJ. (1992). Enzyme studies of methotrexate-resistant human leukemic cell (K562) subclones. *Leukemia Res.*, **16**, 565-569.

LI WW, WALTHAM M, TONG W, SCHWEITZER BI AND BERTINO JR. (1993). Increased activity of $\gamma$-glutamyl hydrolase in human sarcoma cell lines: a novel mechanism of intrinsic resistance to methotrexate. In *Chemistry and Biology of Pteridines, Advances in Experimental Medicine and Biology*, Vol. 338, Ayling JE, Nair MG and Baugh CM. (eds) pp. 635-638. Plenum Press: New York.

MCBURNEY MW AND WHITMORE GF. (1974). Isolation and Biochemical characterisation of folate-deficient mutants of Chinese Hamster Cells. *Cell*, **2**, 173-182.

MCCLOSKEY DE, MCGUIRE JJ, RUSSELL CA, ROWAN BG, BERTINO JR, PIZZORNO G AND MINI E. (1991). Decreased folylpolyglutamate synthetase activity as a mechanism of methotrexate resistance in CCRF-CEM human leukemia sublines. *J. Biol. Chem.*, 6181-6187.

MARSHAM PR, HUGHES LR, JACKMAN AL, HAYTER AJ, OLDFIELD J, WARDLEWORTH JM, BISHOP JA, O'CONNOR BM AND CALVERT AH. (1991). Quinazoline antifolate thymidylate synthase inhibitors: heterocyclic benzoyl ring modifications. *J. Med. Chem.*, **34**, 1594-1605.

O'CONNOR BM, JACKMAN AL, CROSSLEY PH, FREEMANTLE SE AND CALVERT AH. (1992). Human lymphoblastoid cells with acquired resistance to C2-desamino-C2-methyl-$N^{10}$-propargyl-5,8-dideazafolic acid (ICI 198583): a novel folate-based TS inhibitor. *Cancer Res.*, **52**, 1137-1143.

PAVLOVIC M, LEFFERT JJ, RUSSELLO O, BUNNI MA, BEARDSLEY GP, PRIEST DG AND PIZZORNO G. (1993). Altered transport of folic acid and antifolates through the carrier-mediated reduced folate transport system in a human leukemia cell line resistant to (6R) 5,10-dideazatetrahydrofolic acid (DDATHF). In *Advances in Experimental Medicine and Biology*, Vol. 338, Ayling JE, Nair MG and Baugh CM. (eds) pp. 775-778. Plenum Press: New York.

PIALL EM, CURTIN NJ, AHERNE GW, HARRIS AL AND MARKS V. (1989). The quantitation by radioimmunoassay of 2'deoxyuridine-5'-triphosphate in extracts of thymidylate synthase inhibited cells. *Anal. Biochem.*, **177**, 347-352.

PIZZORNO G, MINI E, CORONNELLO M, MCGUIRE JJ, MOROSON BA, CASHMORE AR, DREYER RN, LIN JT, MAZZEI T, PERITI P AND BERTINO JR. (1988). Impaired polyglutamation of methotrexate as a cause of resistance in CCRF-CEM cells after short-term, high-dose treatment with this drug. *Cancer Res.*, **48**, 2149-2155.

PIZZORNO G, CHANG Y-M, MCGUIRE JJ AND BERTINO JR. (1989). Inherent resistance of human squamous carcinoma cell lines to methotrexate as a result of decreased polyglutamation of this drug. *Cancer Res.*, **49**, 5275-5280.

PRIEST DG, LEDFORD BE AND DOIG MT. (1980). Increased thymidylate synthetase in 5-fluorodeoxyuridine-resistant cultured hepatoma cells. *Biochem. Pharmacol.*, **29**, 1549-1553.

RAYNAUD F, HARDCASTLE A AND AHERNE W. (1993). Sensitive measurement of dUMP by radioimmunoassay. *Br. J. Cancer*, **67** (Suppl. XX), 62.

RHEE MS, WANG Y, NAIR MG AND GALIVAN J. (1993). Acquisition of resistance to antifolates caused by enhanced $\gamma$-glutamyl hydrolase activity. *Cancer Res.*, **53**, 2227-2230.

ROSSANA C, RAO LG AND JOHNSON LF. (1982). Thymidylate synthase overproduction in 5-fluorodeoxyuridine-resistant mouse fibroblasts. *Mol. Cell. Biol.*, **2**, 1118-1125.

SAMUELS LL, GOUTAS LJ, PRIEST DG, PIPER JR AND SIROTNAK FM. (1886). Hydrolytic cleavage of methotrexate $\gamma$-polyglutamates by folylpolyglutamyl hydrolase derived from various tumours and normal tissues of the mouse. *Cancer Res.*, **46**, 2230-2235.

SANGHANI PC, JACKMAN A, EVANS VR, THORNTON T, HUGHES L, CALVERT AH AND MORAN RG. (1994). A strategy for the design of membrane-permeable folyl-γ-glutamate synthetase inhibitors: 'Bay-region'-substituted 2-desamino-2-methyl-5,8-dideazafolate analogs. *Mol. Pharmacol.*, **45**, 341-351.
SIKORA E, JACKMAN AL, NEWELL DR AND CALVERT AH. (1988). Formation and retention and biological activity of $N^{10}$-propargyl-5,8-dideazafolic acid (CB3717) polyglutamates in L1210 Cells *in vitro*. *Biochem. Pharmacol.*, **37**, 4047-4054.
SKELTON LA, KIMBELL R, BRUNTON LA, BOYLE FT AND JACKMAN AL (1994). 2-pyridyl quinazolines as inhibitors of thymidylate synthase. *Proc. Am. Assoc. Cancer Res.*, **35**, 301.
SMITH, IE, SPIELMAN M, BONNETERRE J, NAMER M, GREEN M, WANDER HE, TOUSSAINT C AND AZAB M. (1994). Tomudex (ZD1694), a new thymidylate synthase inhibitor with antitumour activity in breast cancer. *Ann. Oncol.*, **5** (Suppl. 5), 132.
SZETO DW, CHENG YC, ROSOWSKY A, YU C-S, MODEST EJ, PIPER JR, TEMPLE C, ELLIOTT RD, ROSE JD AND MONTGOMERY JA. (1979). Human thymidylate synthase-III. *Biochem. Pharmacol.*, **28**, 2633-2637.
VAN DER LAAN BFAM, JANSEN G, KATHMANN I, SCHORNAGEL JH AND HORDIJK GJ. (1991). Mechanisms of acquired resistance to methotrexate in a human squamous carcinoma cell line of the head and neck, exposed to different drug schedules. *Eur. J. Cancer*, **27**, 1274-1278.
WARD WHJ, KIMBELL R AND JACKMAN AL. (1992). Kinetic characteristics of ICI D1694: a quinazoline antifolate which inhibits thymidylate synthase. *Biochem. Pharmacol.*, **43**, 2029-2031.
WEBBER SE, BLECKMAN TM, ATTARD J AND 18 OTHERS. (1993). Design of thymidylate synthase inhibitors using protein crystal structures: the synthesis and biological evaluation of a novel class of 5-substituted quinazolinones. *J. Med. Chem.*, **36**, 733-746.
ZALCBERG J, CUNNINGHAM D, VAN CUTSEM E, FRANCOIS E, SCHORNAGEL JH, ADENIS A, GREEN M, STARKHAMMER H AND AZAB M. (1994). Good antitumour activity of the new thymidylate synthase inhibitor Tomudex (ZD1694) in colorectal cancer. *Ann. Oncol.*, **5** (Suppl. 5), 133.